# A barley stripe mosaic virus‐based guide RNA delivery system for targeted mutagenesis in wheat and maize

**DOI:** 10.1111/mpp.12849

**Published:** 2019-07-05

**Authors:** Jiacheng Hu, Shaoya Li, Zhaolei Li, Huiyuan Li, Weibin Song, Haiming Zhao, Jinsheng Lai, Lanqin Xia, Dawei Li, Yongliang Zhang

**Affiliations:** ^1^ State Key Laboratory of Agro‐Biotechnology and Ministry of Agriculture Key Laboratory of Soil Microbiology, College of Biological Sciences China Agricultural University Beijing 100193 China; ^2^ Institute of Crop Sciences Chinese Academy of Agricultural Sciences Beijing 100081 China; ^3^ State Key Laboratory of Agrobiotechnology and National Maize Improvement Center, Department of Plant Genetics and Breeding China Agricultural University Beijing 100193 China

**Keywords:** *Barley stripe mosaic virus*, CRISPR mutagenesis, delivery, gRNA, maize, *Nicotiana benthamiana*, targeted mutagenesis, wheat

## Abstract

Plant RNA virus‐based guide RNA (gRNA) delivery has substantial advantages compared to that of the conventional constitutive promoter‐driven expression due to the rapid and robust amplification of gRNAs during virus replication and movement. To date, virus‐induced genome editing tools have not been developed for wheat and maize. In this study, we engineered a barley stripe mosaic virus (BSMV)‐based gRNA delivery system for clustered regularly interspaced short palindromic repeat (CRISPR)/Cas9‐mediated targeted mutagenesis in wheat and maize. BSMV‐based delivery of single gRNAs for targeted mutagenesis was first validated in *Nicotiana benthamiana*. To extend this work, we transformed wheat and maize with the Cas9 nuclease gene and selected the wheat *TaGASR7* and maize *ZmTMS5* genes as targets to assess the feasibility and efficiency of BSMV‐mediated mutagenesis. Positive targeted mutagenesis of the *TaGASR7* and *ZmTMS5* genes was achieved for wheat and maize with efficiencies of up to 78% and 48%. Our results provide a useful tool for fast and efficient delivery of gRNAs into economically important crops.

## Introduction

Clustered regularly interspaced short palindromic repeat (CRISPR)/Cas9 systems that defend against invasive pathogens are prevalent in both bacteria and Archaea (Bhaya *et al*., 2011). The most widely used CRISPR/Cas9 system for molecular genetics was derived from the adaptive immunity system of *Streptococcus pyogenes* and consists of the endonuclease Cas9 and partly paired short RNA, crRNA and trans‐crRNA sequences that can be engineered into a single guide RNA (gRNA) (Jinek *et al*., 2012). The gRNA retains the Cas9 interaction structure and the ability to recognize target genes. In plants, gRNAs direct the Cas9 protein to cut double‐stranded target DNAs in cells to create double‐strand breaks that can be repaired by the error‐prone non‐homology end joining pathway and/or the homology‐directed repair pathway to modify or mutate the target genes. The great convenience and versatility provided by the CRISPR/Cas9 system permits broad applications for plant genomic research and plant improvement of economically important crops like wheat (Zhang *et al*., 2016), maize (Li *et al*., 2017) and rice (Sun *et al*., 2016).

Recent studies indicate that viruses can be developed to deliver gRNAs for targeted mutagenesis in plants (Ali *et al*., 2015, 2018; Cody *et al*., 2017; Gil‐Humanes *et al*., 2017; Jiang *et al*., 2019; Yin *et al*., 2015). Compared to the traditional gRNA delivery methods via *Agrobacterium* transformation, plant virus‐mediated gRNA delivery systems have several advantages: (1) the gRNAs can accumulate to high levels owing to viral replication and systemic spread in plants and may contribute to a higher genome editing efficiency, (2) multiple functional gRNAs can be expressed from a single viral genome, which provides the potential for multi‐targeted genome editing, (3) phenotypic alterations may appear in infected plants in a relatively short period of time after gene targeting by virus‐induced genome editing (VIGE), and (4) transformation and regeneration of agriculturally important crops such as wheat is laborious and time‐consuming, but VIGE may shorten this period and simplify operation and editing processes of a target gene in the specific tissues. Several plant RNA viruses including tobacco rattle virus (TRV) (Ali *et al*., 2015), pea early‐browning virus (PEBV) (Ali *et al*., 2018), tobacco mosaic virus (TMV) (Cody *et al*., 2017) and beet necrotic yellow vein virus (BNYVV) (Jiang *et al*., 2019) have been reported to enable targeted genome editing in the model plants *Nicotiana benthamiana* and *Arabidopsis thaliana* or both. The DNA virus cabbage leaf curl virus (CaLCuV) has also been engineered for plant genome editing in tobacco (Yin *et al*., 2015). Moreover, geminivirus viral replicons derived from bean yellow dwarf virus (BeYDV) and wheat dwarf virus (WDV) have been engineered for gene targeting in potato (Butler *et al*., 2016), wheat (Gil‐Humanes *et al*., 2017), rice (Wang *et al*., 2017) and tomato (Dahan‐Meir *et al*., 2018). However, the absence of the viral movement elements in these DNA replicons obviates the spread of these replicons and requires *Agrobacterium*‐mediated transformation or bombardment of the replicons into plant tissues. In contrast, agroinfiltration or mechanical inoculation can be employed for VIGE. To our knowledge, there are currently no VIGE tools for economically important crops like wheat and maize.

**Figure 1:**
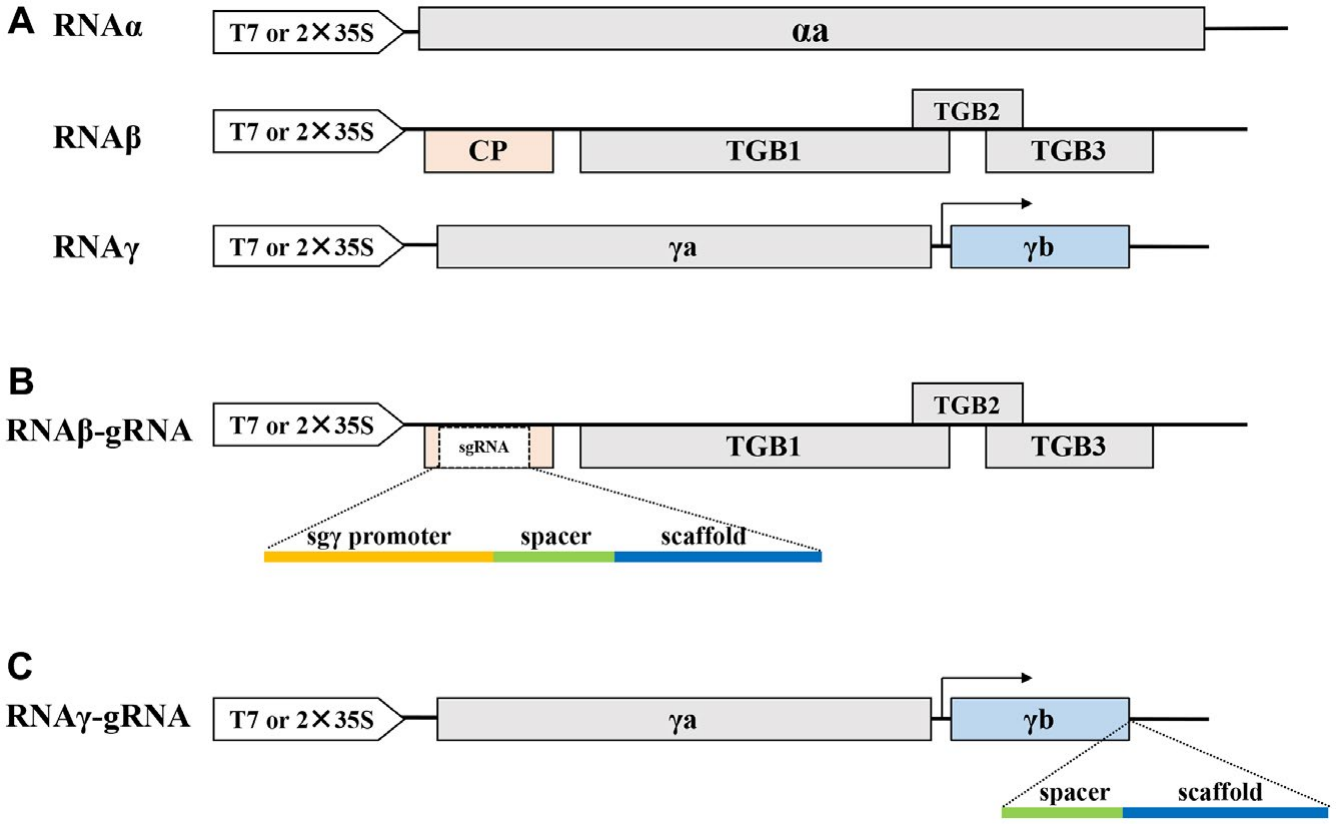
Schematic representation of BSMV‐derived constructs used in this study. (A) BSMV genome organization. The αa protein encoded by RNAα and the γa protein encoded by RNAγ form the replicase of BSMV. RNAβ encodes the coat protein (CP) and triple gene block (TGB) movement proteins. γb, which is translated from subgenomic RNAγ (sgRNAγ), is the pathogenicity determinant and a viral suppressor of RNA silencing (VSR). The arrow indicates the transcription start site of sgRNAγ. (B) ge‐BSMVβ genome organization. Part of the CP sequence (nt 74‐393) is replaced with a 192 nt sgRNAγ promoter (yellow), a gRNA consisting of a 5**'**‐end spacer sequence (green) and the conserved scaffold (blue). (C) ge‐BSMVγ genome organization. A gRNA is inserted downstream of the γb stop codon.

Barley stripe mosaic virus (BSMV) is a single‐stranded RNA virus that has a tripartite RNA genome composed of RNAs α, β and γ (Fig. 1A). In our previous work, we developed BSMV as a high‐throughput virus‐induced gene silencing (VIGS) vector for knock‐down of target genes in monocots and dicots (Yuan *et al*., 2011). Here, we describe a novel BSMV‐based gRNA delivery system (ge‐BSMV) for targeted mutagenesis of the model dicot *N. benthamiana* and the monocot crops wheat and maize.

## Results

### Design of BSMV‐based gRNA delivery systems

To evaluate whether BSMV could be developed as a gRNA delivery tool and used for targeted mutagenesis in plants, we first used the *N. benthamiana* to analyse the feasibility of BSMV‐mediated genome editing. Two different systems engineered by modification of BSMV (ND18 strain) RNAs were designed for expression of gRNAs. The first system, engineered by modification of RNAβ, was designated ge‐BSMVβ and consisted of RNAα, RNAγ and RNAβ‐gRNA (Fig. 1B). A second system, designated ge‐BSMVγ, was based on an RNAγ modification and consisted of RNAα, RNAβ and RNAγ‐gRNA (Fig. 1C). To construct the ge‐BSMVβ system, the 192 nt subgenomic RNAγ (sgRNAγ) promoter that spans nt 1864‐2055 of RNAγ was cloned to isolate the sgRNAγ promoter required for transcription of BSMV sgRNAγ (Johnson *et al*., 2003). Then, the 192 nt sgRNAγ promoter fragment was fused to a 103 nt gRNA ‘spacer scaffold sequence’ to generate a BSMV sgRNAγ promoter‐driven gRNA. The ‘spacer scaffold sequence’ consists of a variable 20 nt spacer designed to pair with the target sequence and the 76 nt gRNA scaffold sequence followed by seven extra downstream T residues. Finally, nt 74 to 393 within the BSMV coat protein open reading frame (ORF) was deleted and replaced with the sgRNAγ promoter‐driven gRNA to generate RNAβ‐gRNA (Fig. 1B). To generate the ge‐BSMVγ system, the ‘spacer scaffold sequence’ was inserted downstream of the γb ORF stop codon to generate the RNAγ‐gRNA (Fig. 1C and Supplemental File S1).

### ge‐BSMV‐mediated targeted mutagenesis in *N*. *benthamiana*

**Figure 2:**
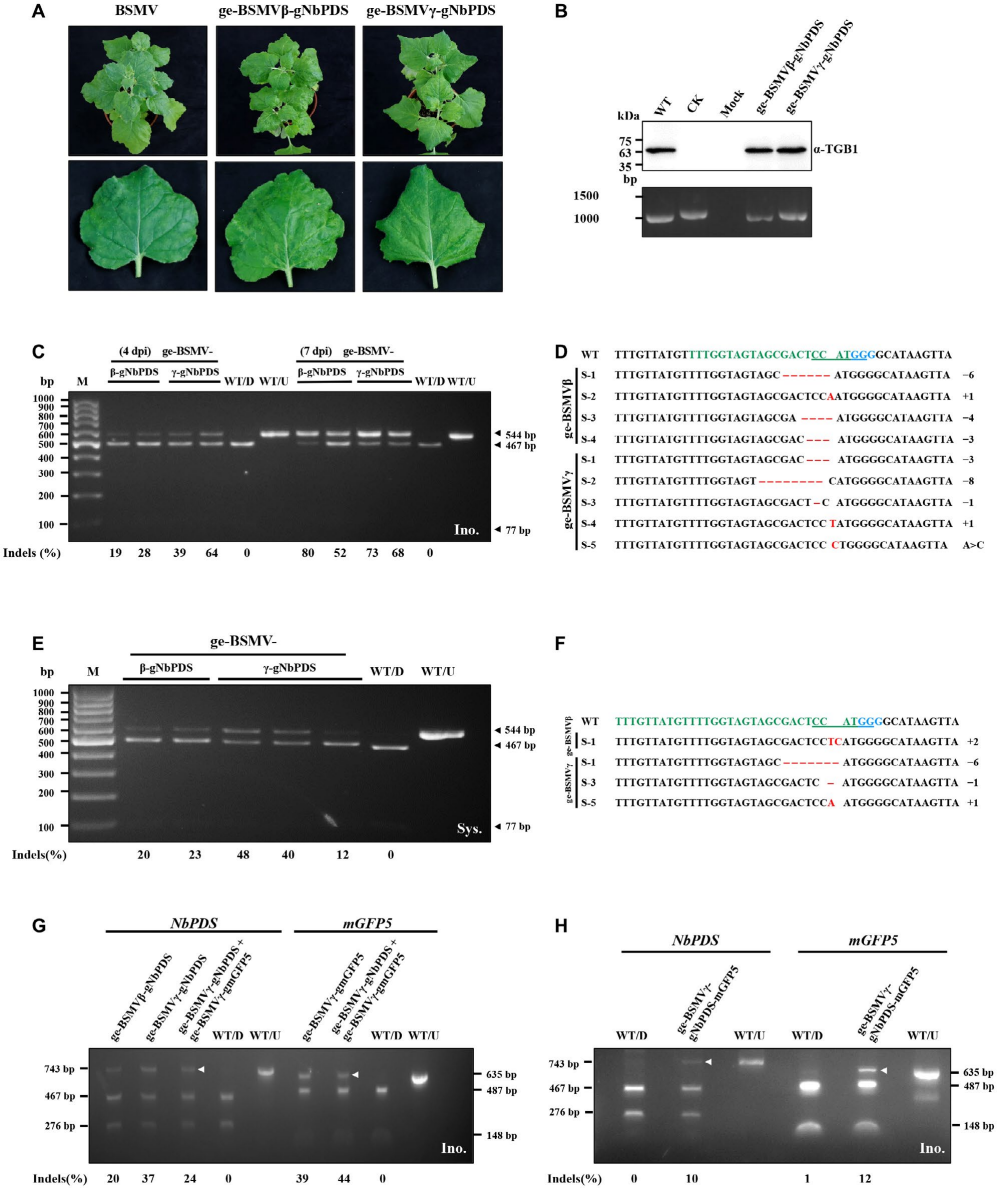
BSMV‐mediated targeted mutagenesis in *Nicotiana benthamiana*. (A) Symptom observations of BSMV and ge‐BSMV‐infiltrated *N. benthamiana*. (B) Western blot and RT‐PCR analysis of ge‐BSMVβ‐gNbPDS and ge‐BSMVγ‐gNbPDS infections in systemically infected *N. benthamiana* leaves. Sample designations are shown above the panels. Antibodies used for the western blot are indicated on the right of the panel. The molecular weight size markers in kDa or the size of DNA fragments in bp are positioned at the left of each panel. WT, BSMV‐infected *N. benthamiana* leaves; CK, pCB301‐BSMVγ‐gNbPDS plasmid as a positive control for evaluating the size of the RT‐PCR products whereas the protein loading buffer alone was used in the corresponding western blot analysis; Mock, empty agrobacterium‐infiltrated *N. benthamiana* plants. (C) PCR/RE analysis of ge‐BSMVβ and ge‐BSMVγ‐mediated genome editing of *NbPDS* in infiltrated *N. benthamiana* leaves at 4 and 7 days post‐infiltration (dpi). The sizes of DNA fragments in bp are indicated at the right of each panel. Arrowheads show the sizes of the target fragments. Mutation frequencies [indels (%)] were calculated by measuring band intensities with ImageJ software (v. 1.51k) and are shown below the corresponding lanes. WT/U and WT/D, DNA fragments derived from wild‐type BSMV infected *N. benthamiana* plants prior to or after *Nco*I digestion; Ino., infiltrated leaves of *N. benthamiana*; M, 100 bp DNA ladder. (D) Sanger sequencing analysis of the *NbPDS* target sites in the DNA samples from ge‐BSMVβ and ge‐BSMVγ‐infiltrated leaves. The protospacer is shown in green, the PAM (NGG) motif is in blue, underlined nucleotides indicate the *Nco*I recognition site, indels are shown in red, with dots indicating deleted nucleotides and red letters indicating inserted nucleotides, the numbers on the right show how many nucleotides were deleted (−) or inserted (+) in the *NbPDS* target site by BSMV‐mediated genome editing. (E) and (F) PCR/restriction enzyme and Sanger sequencing analysis of ge‐BSMVβ and ge‐BSMVγ‐mediated targeted genome editing of *NbPDS* in systemically infected leaves of *N. benthamiana* at 4 dpi. Sys., systemically infected leaves. (G) PCR/RE analysis of ge‐BSMVγ‐mediated multiplexed genome editing in *N. benthamiana* leaves co‐infiltrated with the ge‐BSMVγ‐gNbPDS, ge‐BSMVγ‐gmGFP5 and the Cas9 expression cassette. Two RNAγ‐gRNA‐derived vectors, pCB301‐BSMVγ‐gNbPDS and pCB301‐BSMVγ‐gmGFP5, which contain different gRNAs, were co‐infiltrated with the pCB301‐BSMVα, pCB301‐BSMVβ and Cas9 expression cassettes into *N. benthamiana* line 16C plants. Genomic DNA was extracted from the inoculated leaves at 4 dpi, and DNA fragments flanking the *NbPDS* target or the *mGFP5* target were amplified and digested with *Nco*I or *Nde*I. Arrowheads indicate the *Nco*I‐ or *Nde*I‐resistant bands. The sizes of DNA fragments in bp are indicated at the left (for *NbPDS* fragments) and right (for *mGFP5* fragments) of each panel. WT/D and WT/U, DNA fragments amplified from wild‐type BSMV‐infected *N. benthamiana* plants were digested (D) or not digested (U) with *Nco*I or *Nde*I; Ino., inoculated leaves. (H) PCR/RE analysis of ge‐BSMVγ‐mediated multiplexed genome editing in *N. benthamiana* leaves co‐infiltrated with the ge‐BSMVγ‐gNbPDS‐mGFP5 and Cas9 expression cassettes. Arrowheads indicate the *Nco*I and *Nde*I‐resistant bands. The sizes of the DNA fragments in bp are indicated at the left for the *NbPDS* fragments and right for the *mGFP5* fragments in each panel.

To investigate the feasibility of ge‐BSMVβ and ge‐BSMVγ systems for gene editing, we first tested the two systems in *N*. *benthamiana* by selecting the *NbPDS* gene for targeted editing (Supplemental Table S1). For easy manipulation of the plasmid, full‐length BSMV RNAα, RNAβ and RNAγ were cloned separately into the pCB301‐2X35S‐MCS‐HDV_RZ_NOS plasmid (Yao *et al*., 2011) to generate pCB301‐BSMVα, pCB301‐BSMVβ and pCB301‐BSMVγ. DNA fragments corresponding to the *NbPDS*‐specific gRNA were then cloned into the RNAβ‐gRNA and RNAγ‐gRNA, respectively, as shown in Fig. 1B,C to generate the pCB301‐BSMVβ‐gNbPDS and pCB301‐BSMVγ‐gNbPDS derivatives (see Supplemental File S1). The ge‐BSMV systems consisting of pCB301‐BSMVα/pCB301‐BSMVβ‐gNbPDS/pCB301‐BSMVγ or pCB301‐BSMVα/pCB301‐BSMVβ/pCB301‐BSMVγ‐gNbPDS were designated ge‐BSMVβ‐gNbPDS and ge‐BSMVγ‐gNbPDS. *Agrobacterium* cultures containing different ge‐BSMV components were mixed with *Agrobacterium* harbouring the Cas9 expression vector pHSE401 (Xing *et al*., 2014) and then co‐infiltrated into wild‐type *N*. *benthamiana* leaves. The expression of Cas9 in the infiltrated leaves was confirmed by western blot analysis at 5 days post‐infiltration (dpi) (Supplemental Fig. S1). About 10 days after infiltration, symptoms characterized by downward curved and chlorosis in the leaves were observed in the upper uninoculated leaves of both ge‐BSMVβ‐gNbPDS‐ and ge‐BSMVγ‐gNbPDS‐infiltrated *N. benthamiana* plants (Fig. 2A). Western blot analysis confirmed the presence of TGB1 protein in the systemic leaves (Fig. 2B, upper panel). Furthermore, total RNAs extracted from the systemically infected upper leaves and the region encompassing the inserted gRNA fragment were analysed by RT‐PCR and the results revealed that the size of the RT‐PCR product derived from ge‐BSMVγ‐gNbPDS‐infected leaves was identical to that from the plasmid control and larger than that from the wild‐type BSMV‐infected leaves (Fig. 2B, bottom panel). These results indicate that the ge‐BSMV systems retain the ability to infect *N. benthamiana* systemically and the heterologous gRNA fragment was maintained during systemic infection.

Total RNA from the leaves shown in Fig. 2A were extracted and RT‐qPCR was conducted to analyse the corresponding mRNA levels in these leaves. The results showed that the *NbPDS* mRNA levels in either ge‐BSMVβ‐gNbPDS‐ or ge‐BSMVγ‐gNbPDS‐infiltrated *N. benthamiana* plants were similar to those of the wild‐type BSMV‐infiltrated plants, indicating that the symptoms that appeared in *N. benthamiana* were not due to the VIGS (Supplemental Fig. S2).

At 4 and 7 dpi, genome DNAs from the agroinfiltrated leaves were extracted, and a 544 bp DNA fragment flanking the *NbPDS* target was PCR amplified using the genomic DNA as the template. Because the *NbPDS* target contains a *Nco*I restriction site, editing of the *NbPDS* could be validated by *Nco*I‐digestion of the amplified PCR fragment. Gel electrophoresis of the digested products showed that a *Nco*I‐resistant band was present in the samples from both the ge‐BSMVβ‐gNbPDS and the ge‐BSMVγ‐gNbPDS‐infiltrated leaves (Fig. 2C), suggesting that insertions and deletions (indels) occurred within the *NbPDS* target gene. Sanger sequencing further revealed different indels in the *NbPDS* gene (Fig. 2D). To test whether the ge‐BSMV systems allowed targeted gene editing in the systemically infected leaves of the wild‐type *N. benthamiana* plants infiltrated with ge‐BSMVβ‐gNbPDS or ge‐BSMVγ‐gNbPDS, an *Agrobacterium* culture harbouring the Cas9 expression vector was infiltrated into the systemically infected leaves at 14 dpi. After an additional 4 days, the *NbPDS* target was analysed by a PCR/restriction enzyme (PCR/RE) assay followed by Sanger sequencing as described above. These results showed that indels also occurred at the target *NbPDS* site (Fig. 2E,F), indicating that both ge‐BSMVβ and ge‐BSMVγ are suitable for targeted mutagenesis in both local and systemically infected *N*. *benthamiana* leaf tissue in the presence of Cas9. The PCR/RE assay also suggested that ge‐BSMVγ had higher editing efficiency than that of ge‐BSMVβ in the systemically infected leaves (Fig. 2E), therefore the ge‐BSMVγ system was used for subsequent experiments.

We also attempted to deliver multiple gRNAs simultaneously using the ge‐BSMV system. First, *Agrobacterium* cultures containing pCB301‐RNAγ‐gNbPDS or pCB301‐RNAγ‐gmGFP5 were mixed and co‐infiltrated with pCB301‐BSMVα, pCB301‐BSMVβ and pHSE401 into the leaves of GFP‐transgenic *N. benthamiana* line 16C (Ruiz *et al*., 1998). PCR/RE assays showed that both the *NbPDS* and *mGFP5* genes could be targeted simultaneously in the infiltrated leaves (Fig. 2G). Furthermore, we also tried to engineer two gRNAs in tandem with no space between the gRNAs to generate the ge‐BSMVγ‐gNbPDS‐mGFP5, and the results showed that both *NbPDS* and *mGFP5* could be modified simultaneously in the infiltrated leaves as revealed by the presence of restriction enzyme‐resistant bands in gel images (Fig. 2H). These results indicate that ge‐BSMV‐based VIGE is able to mediate multiplex targeting in *N. benthamiana* inoculated leaves.

### ge‐BSMV‐mediated targeted mutagenesis in wheat

**Figure 3:**
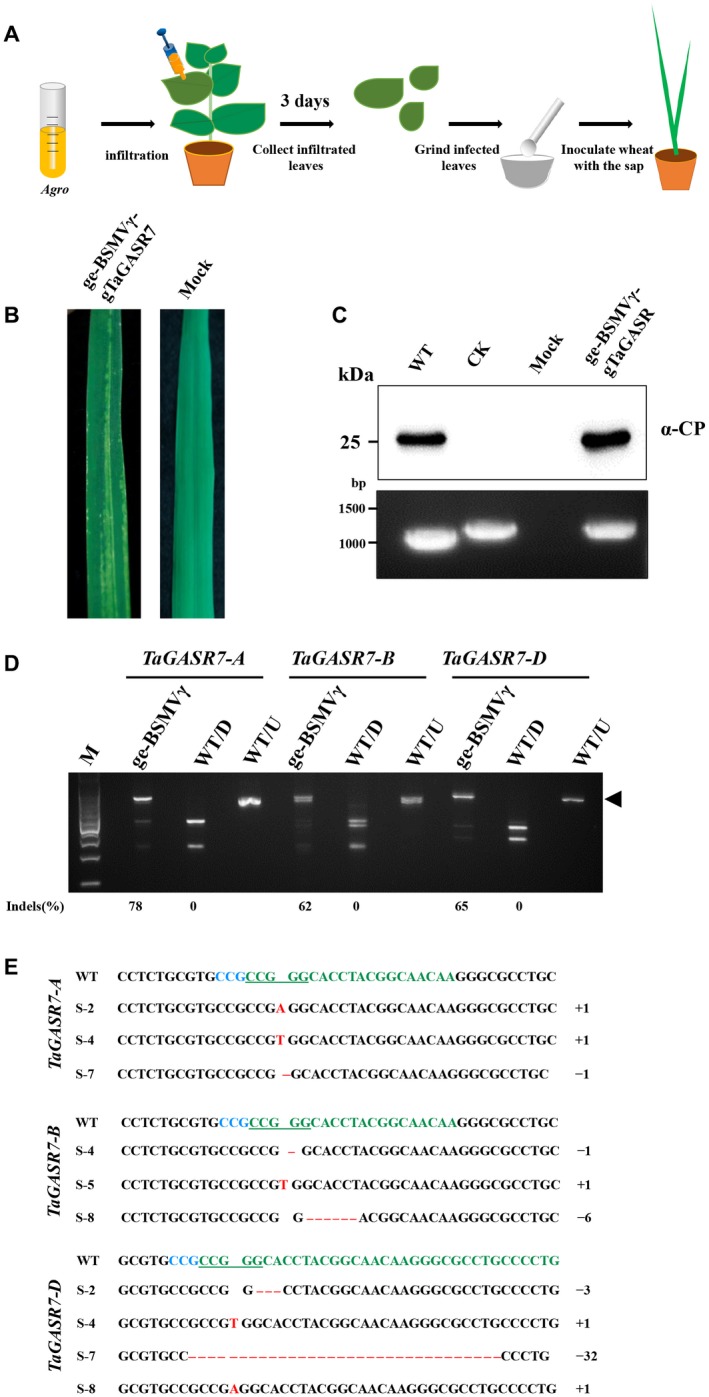
BSMV‐mediated targeted mutagenesis of *TaGASR7* in wheat. (A) Workflow of ge‐BSMV‐mediated editing of target genes in wheat. *Agrobacterium* cultures harbouring the ge‐BSMV plasmids were infiltrated into *Nicotiana benthamiana* leaves, infiltrated leaves were ground at 3 days post‐infiltration (dpi) and the sap was rub‐inoculated onto leaves of Cas9‐transgenic wheat seedlings at the two‐leaf stage. (B) Symptoms of systemically infected wheat leaves at 14 dpi with ge‐BSMVγ‐gTaGASR7. Wheat seedlings rub‐inoculated with sap from empty agrobacterium‐infiltrated *N. benthamiana* leaves served as a control (Mock). (C) Western blot and RT‐PCR analyses of ge‐BSMVγ‐gTaGASR7 systemic infection of wheat. Sample designations are indicated above the panels. Antibodies used for western blots are indicated at the right of the panel. Molecular weight size markers in kDa or the size of DNA fragments in bp are indicated at the left side of each panel. WT, BSMV‐infected wheat leaves; CK, pCB301‐BSMVγ‐gTaGASR7 plasmid used as a positive control for evaluating the size of the RT‐PCR products. The protein loading buffer was used in the western blot controls. (D) PCR/restriction enzyme analysis of ge‐BSMVγ‐mediated genome editing of *TaGASR7* in wheat. Systemically infected leaves showing typical symptoms of BSMV were collected and genomic DNA was extracted. DNA fragments flanking the wheat A, B and D genome targets were amplified and subjected to *Bcn*I digestion. The arrowhead indicates the *Bcn*I‐resistant bands. Mutation frequencies [indels (%)] were calculated by measuring band intensities with ImageJ software (v. 1.51k) and are shown below the corresponding lanes. WT/D and WT/U, *Bcn*I digested (D) or undigested (U) DNA fragments amplified from wild‐type BSMV‐infected wheat. (E) Sanger sequencing of *TaGASR7* target sites in DNA samples extracted from ge‐BSMVγ‐gTaGASR7 infected leaves at 20 dpi. DNA fragments derived from different wheat genomes are indicated on the left. The protospacer is shown in green, the PAM (NGG) motif is in blue, underlined nucleotides indicate the *Bcn*I recognition site, indels are shown in red with dots indicating deleted nucleotides and red letters showing inserted nucleotides. The numbers on the right indicate how many nucleotides are deleted (−) or inserted (+) by BSMV‐mediated genome editing in the *TaGASR7* target site.

As wheat is one of the natural hosts of BSMV, we sought to apply the ge‐BSMVγ system to wheat to provide additional toolkits for targeted mutagenesis in wheat. The *TaGASR7* gene, which is involved in the control of grain length and weight (Zhang *et al*., 2016), was selected as the target (Supplemental Table S1) and the vector pCB301‐BSMVγ‐gTaGASR7 was constructed (see Supplemental File S1). The Cas9 expression cassette was first transformed into common wheat (*Triticum aestivum* 'Zhengmai 7698') and positive lines were confirmed by genomic PCR amplification (Supplemental Fig. S3A). Western blots were also performed to confirm the expression of the Cas9 protein in the transgenic wheat (Supplemental Fig. S3B). *Nicotiana benthamiana* was used as an intermediate host to recover BSMV for wheat infections as described previously (Yuan *et al*., 2011). For this purpose, *N*. *benthamiana* leaves were agroinfiltrated with ge‐BSMVγ‐gTaGASR7, the infiltrated leaves were harvested at 3 dpi and ground in phosphate buffer, and the leaf sap was rubbed on the leaves of Cas9‐transgenic Zhengmai7698 wheat (Fig. 3A). By 14 dpi, typical BSMV symptoms, including chlorotic spots and stripes, appeared on the upper uninoculated leaves (Fig. 3B). Western blot analyses confirmed the presence of the BSMV CP (coat protein) in the leaves (Fig. 3C, upper panel) and RT‐PCR showed that the heterologous gRNA fragment was stable during systemic movement of ge‐BSMVγ‐gTaGASR7 in wheat (Fig. 3C, bottom panel).

About 3 weeks post‐inoculation, genomic DNA was extracted from leaves with symptoms and a DNA fragment flanking the target was amplified from the A, B and D wheat genomes. PCR/RE assays revealed indels in all of the A, B, D genome targets with mutation efficiencies of up to 78% (Fig. 3D). Sanger sequencing further confirmed that diverse *TaGASR7* indels were present in the A, B, and D genomes (Fig. 3E and Supplemental Fig. S4). In addition, *TaGASR7* mRNA accumulation levels in the leaves shown in Fig. 3B were analysed by RT‐qPCR. These results revealed similar *TaGASR7* mRNA levels in both the ge‐BSMVγ‐gTaGASR7‐ and mock‐inoculated wheat, excluding the possibility that these symptoms resulted from VIGS (Supplemental Fig. S5). Altogether, these results demonstrate that the ge‐BSMV system can direct efficient targeted mutagenesis in common wheat.

### ge‐BSMV‐mediated targeted mutagenesis in maize

**Figure 4:**
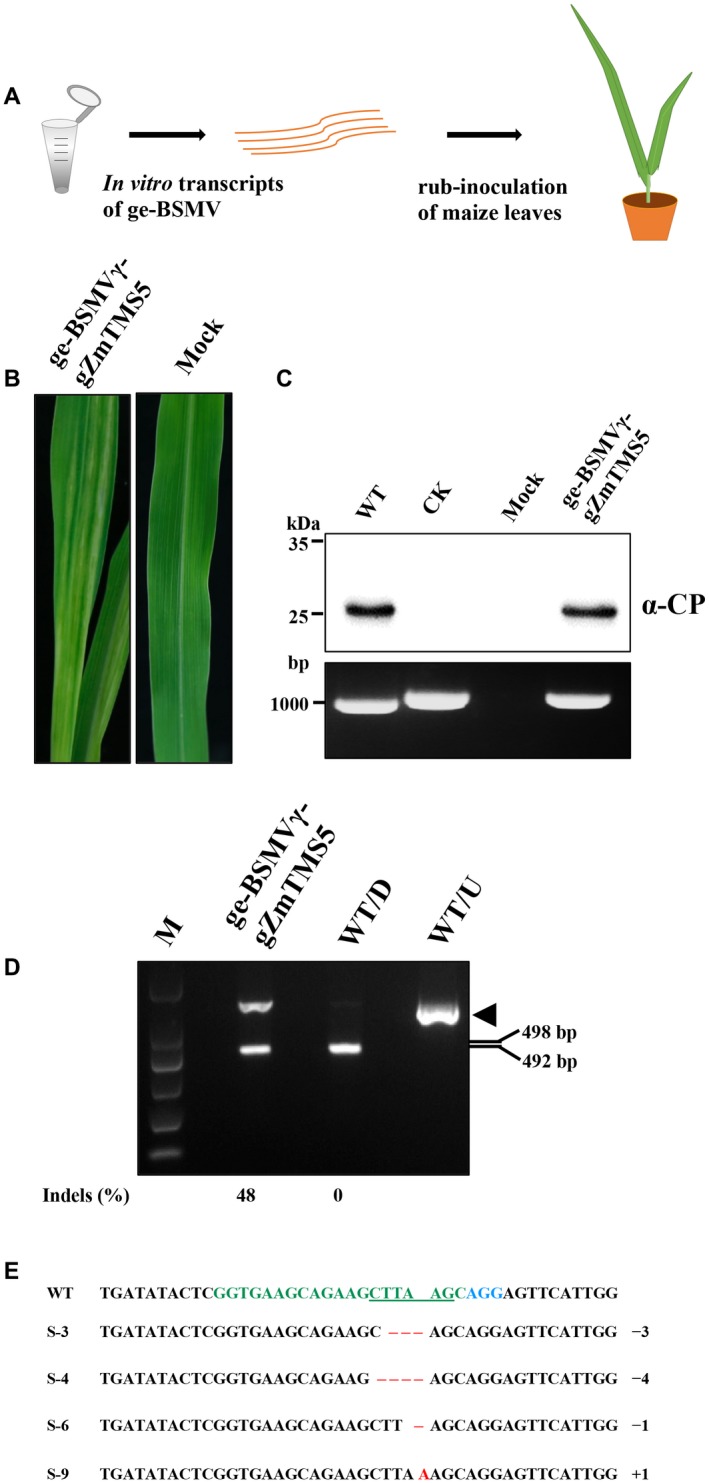
BSMV‐mediated targeted mutagenesis of *ZmTMS5* in maize. (A) Workflow of ge‐BSMV‐mediated targeted gene editing in maize. *In vitro*‐transcripts of pT7‐α_XJ_, pT7‐β_XJ_ and pT7‐γ‐gZmTMS5 were rub‐inoculated onto Cas9‐transgenic maize seedlings at the two‐leaf stage. (B) Symptoms of maize infected with ge‐BSMVγ‐gZmTMS5. Controls were FES buffer‐inoculated maize (Mock). (C) Western blot and RT‐PCR analysis of ge‐BSMVγ‐gZmTMS5 infection in the systemically infected maize leaves. Sample names are indicated above the panels. Antibodies used for western blots are indicated at the right of the panel. The molecular weight size markers in kDa or the sizes of DNA fragments in bp are indicated at the left side of each panel. WT, wild‐type BSMV‐infected maize leaves; CK, the pT7‐γ‐gZmTMS5 plasmid was used as a positive control for evaluating the size of the RT‐PCR products. The protein loading buffer alone was used as a control in western blot analyses. (D) PCR/restriction enzyme analysis of ge‐BSMVγ‐mediated genome editing of *ZmTMS5* in systemically infected maize leaves at 24 days post‐inoculation (dpi). DNA fragments flanking the target site were amplified and subjected to *Afl*II digestion. The arrowhead indicates the *Afl*II‐resistant band (Note: due to size similarities, two of the digested DNA fragments appeared as one band in the gel). Mutation frequencies [indels (%)] were calculated by measuring band intensities with ImageJ software (v. 1.51k) and are shown below the corresponding lanes. WT/D and WT/U, DNA fragments amplified from wild‐type BSMV‐infected maize plants were digested (D) or not digested (U) with *Afl*II. (E) Sanger sequencing analysis of the *ZmTMS5* target site in DNA of leaf samples infected with ge‐BSMVγ‐gZmTMS5 at 24 dpi. The protospacer is shown in green, the PAM (NGG) motif is in blue, underlined nucleotides indicate the *Afl*II recognition site, indels are shown in red with the dots indicating deleted nucleotides and red letters specifying inserted nucleotides. The numbers on the right indicate how many nucleotides were deleted (−) or inserted (+) in the *ZmTMS5* target site by BSMV‐mediated genome editing.

We attempted to apply the ge‐BSMV system to maize, another agriculturally important crop. The Cas9 expression cassette was transformed into maize (*Zea mays* subsp. *mays* 'B73‐329') and positive lines were confirmed by genomic PCR (Supplemental Fig. S6A). Western blots were also performed to confirm the expression of Cas9 protein in the transgenic maize (Supplemental Fig. S6B). The *ZmTMS5* (*thermosensitive genic male‐sterile 5*) gene involved in pollen fertility (Li *et al*., 2017) was selected as the target (Supplemental Table S1), Cas9‐transgenic maize leaves were rub‐inoculated with a mixture of the *in vitro* transcripts of RNAα, RNAβ and RNAγ‐gZmTMS5 (see Supplemental File S1) derived from BSMV Xinjiang (BSMV_XJ_), a maize‐infecting strain (Hu *et al*., 2015)_._ Large‐scale chlorosis and stripes appeared on the newly emerged leaves (Fig. 4B) and western blot analysis revealed the presence BSMV CP in the upper uninoculated leaves (Fig. 4C, upper panel). RT‐PCR analysis further confirmed the maintenance of heterologous gRNA fragments during systemic infection of ge‐BSMVγ‐gZmTMS5 in maize (Fig. 4C, bottom panel).

About 3 weeks post‐inoculation, genomic DNA was extracted from systemically infected leaves exhibiting typical symptoms. A 994 bp DNA fragment flanking the *ZmTMS5* target site was then PCR amplified by using the extracted DNA as the template and a PCR/RE assay was performed to evaluate the mutation rates within the *ZmTMS5* target site. The results showed that an *Afl*II‐resistant band was present only in the samples from ge‐BSMVγ‐gZmTMS5‐inoculated maize, but not from wild‐type BSMV infected maize (Fig. 4D), suggesting that indels occurred within the targeted gene *ZmTMS5*. Moreover, quantitative analyses of the *Afl*II‐digested products indicated mutation efficiencies of up to 48% (Fig. 4D). Sanger sequencing further revealed diverse indels within the *ZmTMS5* target site (Fig. 4E and Supplemental Fig. S7). These results indicate that ge‐BSMV‐based VIGE results in efficient targeted mutagenesis in maize.

## Discussion

Currently, four RNA viruses have been developed as VIGE vectors. Two tobraviruses, TRV and PEBV, can be used to edit *N. benthamiana* and *Arabidopsis* genes of interest (Ali *et al*., 2015, 2018). TMV was also reported to be able to mediate target gene editing in the local leaves by partially substituting the CP ORF with a gRNA (Cody *et al*., 2017). Very recently, BNYVV has also been used for gRNA delivery in *N. benthamiana* (Jiang *et al*., 2019). BSMV‐based VIGE described above provides an alternative tool for targeted gene editing in the model plant *N. benthamiana*. Moreover, the broad host range of BSMV, especially its ability to infect many economically important monocots, enables its potential use as a VIGE tool in several crop plants that TRV, PEBV and TMV are unable to infect.

WDV replicons have been used for genome editing in wheat (Gil‐Humanes *et al*., 2017), but compared to WDV replicons, BSMV‐based VIGE does not require integration of the viral element into the wheat genome, which avoids raising additional regulatory and ethical issues. Although *in vitro* transcripts of CRISPR/Cas9 RNA (Zhang *et al*., 2016) and CRISPR/Cas9 RNP (Liang *et al*., 2017) were used to generate transgene‐free genome editing in wheat, the ratio of positive offspring with targeted mutations is relatively low due to rapid degradation of introduced gRNA by cellular nucleases (Woo *et al*., 2015). In contrast, the ge‐BSMV‐based VIGE tools used in our study showed high efficiencies of targeted gene editing in the leaves of wheat, as illustrated by the large number of gRNA‐containing fragments accompanying BSMV replication, and the protection of gRNA‐containing fragments by viral proteins such as γb silencing suppressor (Bragg *et al*., 2004; Yelina *et al*., 2002; Zhang *et al*., 2017).

To test the inheritance of BSMV‐mediated gene editing in the *N. benthamiana* leaves, we regenerated plants from leaf tissues containing the edited *NbPDS* gene, and several regenerated albino plants were obtained (Supplemental Fig. S8), suggesting that the edited gene can be passed to subsequent generations. Intriguingly, it has been reported that maize seedlings can be regenerated from leaf segments (Lowe *et al*., 2016; Yu *et al*., 2012). Therefore, our BSMV‐based gene editing system may provide an alternative approach to obtain genetically modified maize, which can bypass immature embryo transformation and regeneration processes. Furthermore, BSMV is capable of entering the developing ovules and embryo of barley as well as sperm and vegetative cells of barley pollen (Carroll, 1969, 1974; Carroll and Mayhew, 1976) to result in seed transmissibility of BSMV (Carroll, 1972). Unfortunately, to the best of our knowledge, seed transmission of BSMV in wheat has not been evaluated. Therefore, studies of appropriate cultivars in which the BSMV_ND_ isolate or other BSMV strains can be seed‐transmitted with high efficiency might increase the feasibility of using seed‐borne approaches for VIGE genome editing in wheat.

In addition to wheat and maize, some BSMV strains are able to infect barley (*Hordeum vulgare*) (Jackson *et al*., 2009), *Brachypodium distachyon* (Demircan and Akkaya, 2010; Pacak *et al*., 2010), oat (Pacak *et al*., 2010), millet (*Setaria italic*) (unpublished results), sorghum (*Sorghum bicolor*) (unpublished results) and several other cereals. Our proof‐of‐concept study of BSMV‐based VIGE in *N. benthamiana*, wheat and maize provides the basis for expanded use in other economically important monocot plants.

## Experimental Procedures

### Vector construction

To construct the pCB301‐BSMVα, pCB301‐BSMVβ and pCB301‐BSMVγ, plasmids BSMV RNAα, RNAβ and RNAγ were amplified from pT7‐α_ND_, pT7‐β_ND_ and pT7‐γ_ND_ (Petty *et al*., 1989), respectively, then cloned into *Stu*I‐ and *Bam*HI‐digested pCB301‐2X35S‐MCS‐HDV_RZ_NOS (Yao *et al*., 2011) using a Seamless Assembly Cloning Kit (Clone Smarter Technologies Inc., Houston, USA) according to the manufacturer’s instructions. To construct the pCB301‐BSMVβ‐gNbPDS plasmid, nt 74–393 of the BSMV CP ORF were deleted by reverse PCR using pCB301‐BSMVβ as the template, the sequence encoding the gRNA scaffold was synthesized by GENEWIZ Inc. (GENEWIZ, Inc., South Plainfield, NJ, USA), and the gRNA scaffold sequence along with seven extra downstream T residues was then amplified and cloned into the pCB301‐BSMVβ_Δ74‐393_ using the Seamless Assembly Cloning Kit (Clone Smarter Technologies Inc., Houston, USA). The resulting plasmid was linearized by a second round of reverse PCR, and a 192 bp sgRNAγ promoter corresponding to nt 1864–2055 of BSMV RNAγ was amplified from pCB301‐BSMVγ and cloned into the linearized vector using the same strategy as described above. The 20 bp spacer targeting the *NbPDS* gene sequence was inserted between the sgRNAγ promoter sequence and the sequence encoding the gRNA scaffold by a third round of reverse PCR to generate the pCB301‐BSMVβ‐gNbPDS derivative. To construct the pCB301‐BSMVγ‐gNbPDS, reverse PCR was performed to introduce *Nco*I, *Mlu*I, *Spe*I and *Apa*I restriction sites into the downstream γb ORF sequence to generate the plasmid pCB301‐BSMVγ‐MCS (see Supplemental File S1), and the gRNA sequence was amplified from pCB301‐BSMVβ‐gNbPDS and cloned into pCB301‐BSMVγ‐MCS via the *Nco*I and *Spe*I restriction enzyme sites.

For easy manipulation of the plasmids, a pair of reversely arranged *Sap*I restriction enzyme sites was inserted downstream of the γb ORF by reverse PCR to generate the plasmid pCB301‐BSMVγ‐SapI (see Supplemental File S1). *SmR* gene was amplified from pHSE401 (Xing *et al*., 2014) and inserted between the two *Sap*I sites using the Seamless Assembly Cloning Kit (Clone Smarter Technologies Inc., Houston, USA), resulting in the pCB301‐BSMVγ‐SmR. To construct the pCB301‐BSMVγ‐gTaGASR7, pCB301‐BSMVγ‐SmR was digested by *Sap*I, ligated with annealed oligos with a 5′‐CTA and a 3′‐CAA overhangs. Oligos annealing was conducted according to previously described methods (Shan *et al*., 2014) with minor modifications. Briefly, a pair of 100 μM oligos were 5′ phosphorylated using T4 polynucleotide kinase (Thermo Fisher Scientific, Inc., Pittsburgh, PA, USA) and annealed in the thermal cycler using the following steps: denaturation at 95 °C for 5 min and gradually chilled to 25 °C with 1 °C drops per minute. A final step was performed by maintaining the samples at 16 °C for 10 min.

To construct the RNAγ‐gZmTMS5 vector for *in vitro* transcription, a pCB301‐BSMVγ‐gZmTMS5 plasmid targeting the ZmTMS5 gene was constructed as described above using oligos ZmTMS5‐T2‐F/R. The gRNA sequence was amplified from the pCB301‐BSMVγ‐gZmTMS5 and cloned into pT7‐γ_XJ_ (Hu *et al*., 2015) downstream of the γb stop codon to generate the pT7‐γ‐gZmTMS5 using the Seamless Assembly Cloning Kit (Clone Smarter Technologies Inc., Houston, USA).

To construct the RNAγ‐gNbPDS‐mGFP5, four primers, Fb1, F1, R2 and Rb2, were used to amplify a DNA fragment containing the two different gRNA sequences using pCB301‐BSMVγ‐SmR as the template. The concentration of primers F2 and R2 used for PCR was 1/20 of the Fb1 and Rb2 concentrations. The PCR products were then cloned into *Sap*I‐digested pCB301‐BSMVγ‐SmR by using the seamless cloning procedure described above.

All of the primers used in this study were listed in Supplemental Table S2 and DNA sequencing was conducted to confirm the correctness of these plasmids. The sequence information of the BSMV derivatives used in this study can be found in Supplemental File S1.

### Plant growth conditions

Wheat, maize and *N. benthamiana* plants were grown in a greenhouse under a 14/10 h light/dark photoperiod at 23–25 °C as previously described (Yuan *et al*., 2011). Wheat and maize seedlings were grown to the two‐leaf stage prior to rub‐inoculation, and inoculated plants were maintained in the same climate chamber as described above (Hu *et al*., 2015).

### Transformation of the Cas9 expression cassette into wheat and maize

To construct Cas9‐transgenic wheat, the pCXUN‐Cas9 plasmid was transformed into calli of common wheat (*T. aestivum* 'Zhengmai 7698') by particle bombardment following the protocol described previously (Sun *et al*., 2019).

To create Cas9‐transgenic maize, the Cas9 expression cassette was cloned into the pCAMBIA3301 binary vector followed by transformation into *A. tumefaciens* strain EHA105. *Agrobacterium tumefaciens*‐mediated transformation of maize was carried out as described previously (Zhu *et al*., 2016).

### Agroinfiltration and inoculation of plants with *in vitro* synthesized RNAs

The pCB301‐BSMVα, pCB301‐BSMVβ, pCB301‐BSMVγ, pCB301‐BSMVβ‐gRNA and pCB301‐BSMVγ‐gRNA plasmids were transformed into *A. tumefaciens* EHA105 strains for agroinfiltration of *N. benthamiana* leaves. Equal volumes of *Agrobacterium* strains harbouring individual plasmids were mixed to a final OD_600_ of 0.3 and infiltrated into leaves of 3‐ to 4‐week‐old *N. benthamiana* plants as described previously (Yuan *et al*., 2011). *Agrobacterium* harbouring the Cas9 expression construct was infiltrated at a final OD_600_ of 0.5.

For maize, the BSMV Xinjiang strain (BSMV_XJ_) was used for inoculation. *In vitro* RNA transcripts were mechanically inoculated onto two‐leaf stage maize seedlings as described previously (Hu *et al*., 2015; Lee *et al*., 2012). Briefly, *in vitro* transcripts from pT7‐α_XJ_, pT7‐β_XJ_ and pT7‐γ_XJ_ (or pT7‐γ‐gZmTMS5) were mixed at a molar ratio of 1:1:1. These transcripts were then mixed with equal volumes of FES buffer (0.06 M potassium phosphate, 0.1 M glycine, 1% bentonite, 1% sodium pyrophosphate decahydrate, 1% celite, pH 8.5) followed by rub‐inoculation of maize leaves.

### PCR/RE assay and Sanger sequencing

*Nicotiana benthamiana*, wheat and maize genomic DNA was extracted using the cetyltrimethylammonium bromide (CTAB) method as previously described (Doyle and Doyle, 1987). PCR/RE assay was performed according to previously described methods with minor modifications (Shan *et al*., 2014). Briefly, four to eight pieces of leaves from different inoculated plants were harvested for genomic DNA extraction, and a DNA fragment flanking the target site was amplified using the extracted genomic DNA as a template. The PCR products (200–300 ng) were digested with restriction enzymes corresponding to the target site followed by gel electrophoresis. For Sanger sequencing, 200–300 ng of PCR products were digested with corresponding restriction enzymes, the digested products were TOPO‐cloned into the easy vector using pClone007 Blunt Simple Vector Kit (Cat No. TSV‐007BS, Tsingke, Beijing, China), and 6–10 positive clones were sequenced by GENEWIZ Inc (GENEWIZ, Inc., South Plainfield, NJ, USA).

### RT‐qPCR and statistical analysis

RT‐qPCR was performed as described previously with minor modifications (Liu *et al*., 2012; Zhang *et al*., 2013) Briefly, total RNAs were extracted and quantified by a NanoDrop ND‐1000 (Thermo Fisher Scientific, Inc., Pittsburgh, PA, USA). For RT‐qPCR, cDNA was synthesized from 2 μg of DNase‐treated total RNA using an oligo‐dT primer and M‐MLV reverse transcriptase (Promega, Madison, WI, USA). The gene fragments were amplified using 2 × SsoFast EvaGreen Supermix (Bio‐Rad, Laboratories, Inc., Hercules, CA, USA) with corresponding primers listed in Supplemental Table S2. The expression levels were normalized by reference gene *PP2A* for *NbPDS* or *EF1α* for *TaGASR7*. Values indicate mean ± SD. For *NbPDS*, the quantified data in each panel were subjected to statistical analysis using SPSS software (v. 22.0, IBM) and the data were compared using one‐way analysis of variance (ANOVA). Significant differences in *NbPDS* mRNA accumulation were determined by Duncan’s multiple range test. For *TaGASR7*, significant difference was analysed using Student’s *t*‐test. Values are mean ± SD (ns, not significant, *n* = 3).

## Conflict of Interests

The authors declare no conflict of interests.

## Supporting information


**Fig. S1** Western blot analysis of transiently expressed Cas9 in *N. benthamiana*.

**Fig. S2** RT‐qPCR analysis of *NbPDS* mRNA level in ge‐BSMV infected *N. benthamiana* leaves.

**Fig. S3** Genomic PCR (A) and western blot analysis (B) of Cas9‐transgenic wheat. For western blot analysis, the Cas9‐specific monoclonal antibody (Cat No.#14697, Cell Signaling Technology, Massachusetts, USA) was used at a 1:1000 dilution.

**Fig. S4** Representatives of Sanger sequencing chromatograms of indels from systemically infected wheat leaves. Black lines under the sequence indicates the target site. ‐RC, reverse complement sequence.

**Fig. S5** RT‐qPCR analysis of *TaGASR7* mRNA levels in ge‐BSMV infected wheat leaves. Data are represented as means ± SD (Student’s *t‐*test; ns, not significant; *n* = 3).

**Fig. S6** Genomic PCR (A) and western blot analysis (B) of the Cas9‐transgenic maize. For western blot analysis, Cas9 monoclonal antibody (Cat No.#14697, Cell Signaling Technology) was used with 1:1000 dilution.

**Fig. S7** Representative Sanger sequencing chromatograms showing indels from systemically infected maize leaves. Black lines under the sequence indicates the target site. ‐RC, reverse complement sequence.

**Fig. S8** Regeneration of *Nicotiana benthamiana* leaf segments containing the edited *NbPDS* gene. ge‐BMVSγ‐gNbPDS‐infected systemically infected leaves of* N. benthamiana* were harvested at 30 days post‐inoculation (dpi), surface sterilized with 2.0–2.5% sodium hypochlorite, rinsed three times with sterile water, cut into *c*.2 cm² pieces and placed on medium plates (MS medium, 30.0 g/L sucrose, 1.0 mg/L zeatin, 2.0 mg/L kinetin, 1.0 mg/L indole‐3‐acetic acid, 350.0 mg/L carbenicillin, 4.0 g/L phytagel, pH 5.8) for differentiation under controlled conditions (23 °C, 16 h photoperiod). After 4–6 weeks, shoots with bleaching phenotypes were excised, transferred to rooting medium plates (1/3× MS medium, 7.0 g/L glucose, 3.0 g/L sucrose, 100.0 mg/L carbenicillin, 4.0 g/L phytagel, pH 5.8) and incubated at 23 °C. Representative *N. benthamiana* shoots regenerated from the leaf segments were photographed. Boxed regions in the left panels were magnified to show the regenerated albino plants.

**Table S1** Cas9 targets selected in this study.

**Table S2** Primers used in this study.

**File S1** Sequences of RNAβ‐gNbPDS, RNAγ‐gNbPDS, RNAγ‐gTaGASR7, RNAγ‐gZmTMS5, RNAγ‐gNbPDS‐mGFP5, RNAγ‐SmR and pCB301‐BSMVγ‐MCS. Sequences in green indicate the sgRNAγ promoter, whereas sequences in red, blue and purple represent the gRNA spacer, the scaffold and the SmR gene, respectively. The underlined sequences indicate the *Sap*I recognition sites or the multiple cloning sites.
