# Supplementary figures and images for: A barley stripe mosaic virus‐based guide RNA delivery system for targeted mutagenesis in wheat and maize
Source: Mol Plant Pathol. 2019 Jul 5;20(10):1463–74. doi: 10.1111/mpp.12849 (PMC6792137; doi:10.1111/mpp.12849)

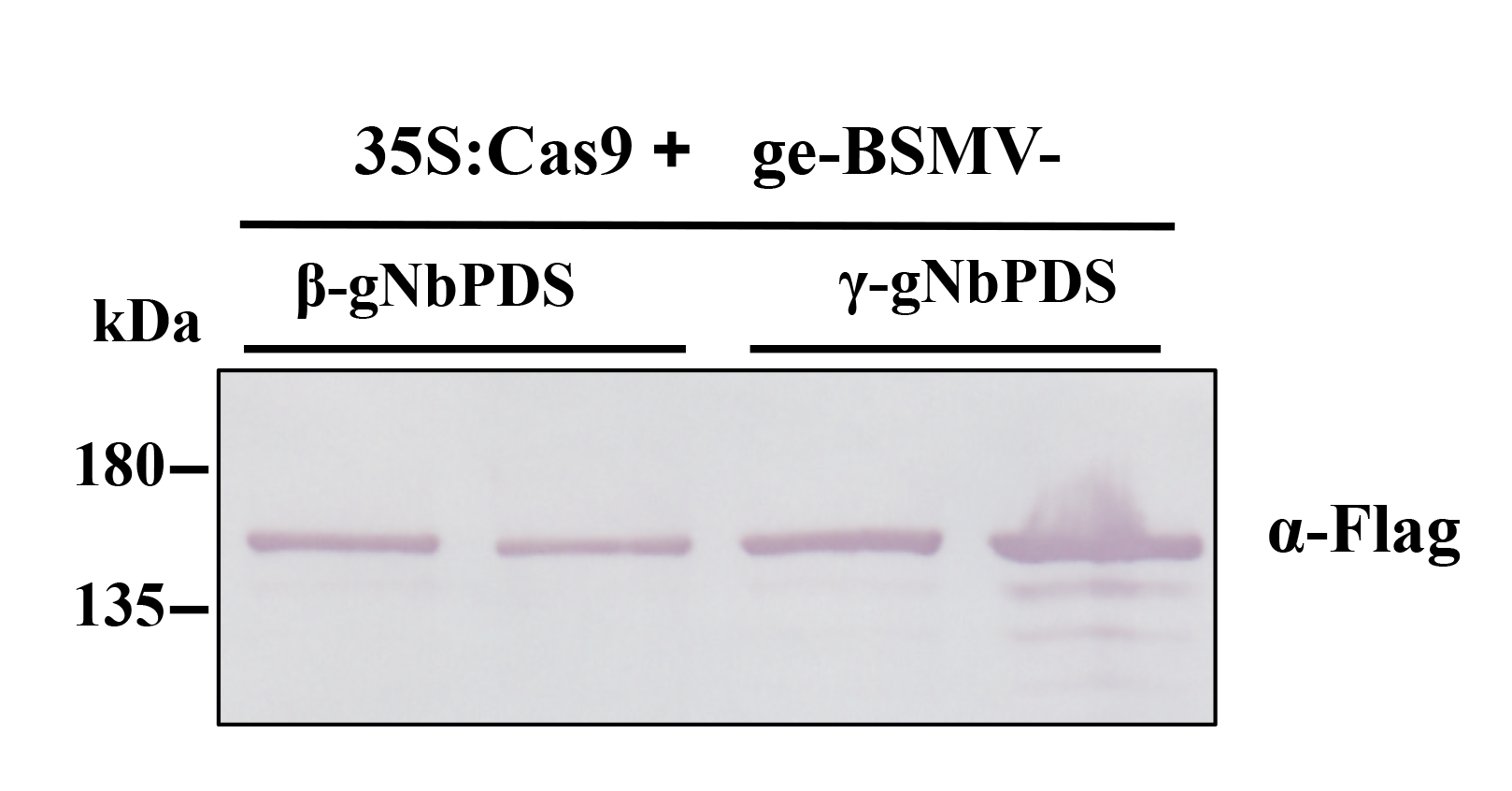

Supplement: Supplementary file 1 — Fig. S1 Western blot analysis of transiently expressed Cas9 in N. benthamiana. [file MPP-20-1463-s001.tif]

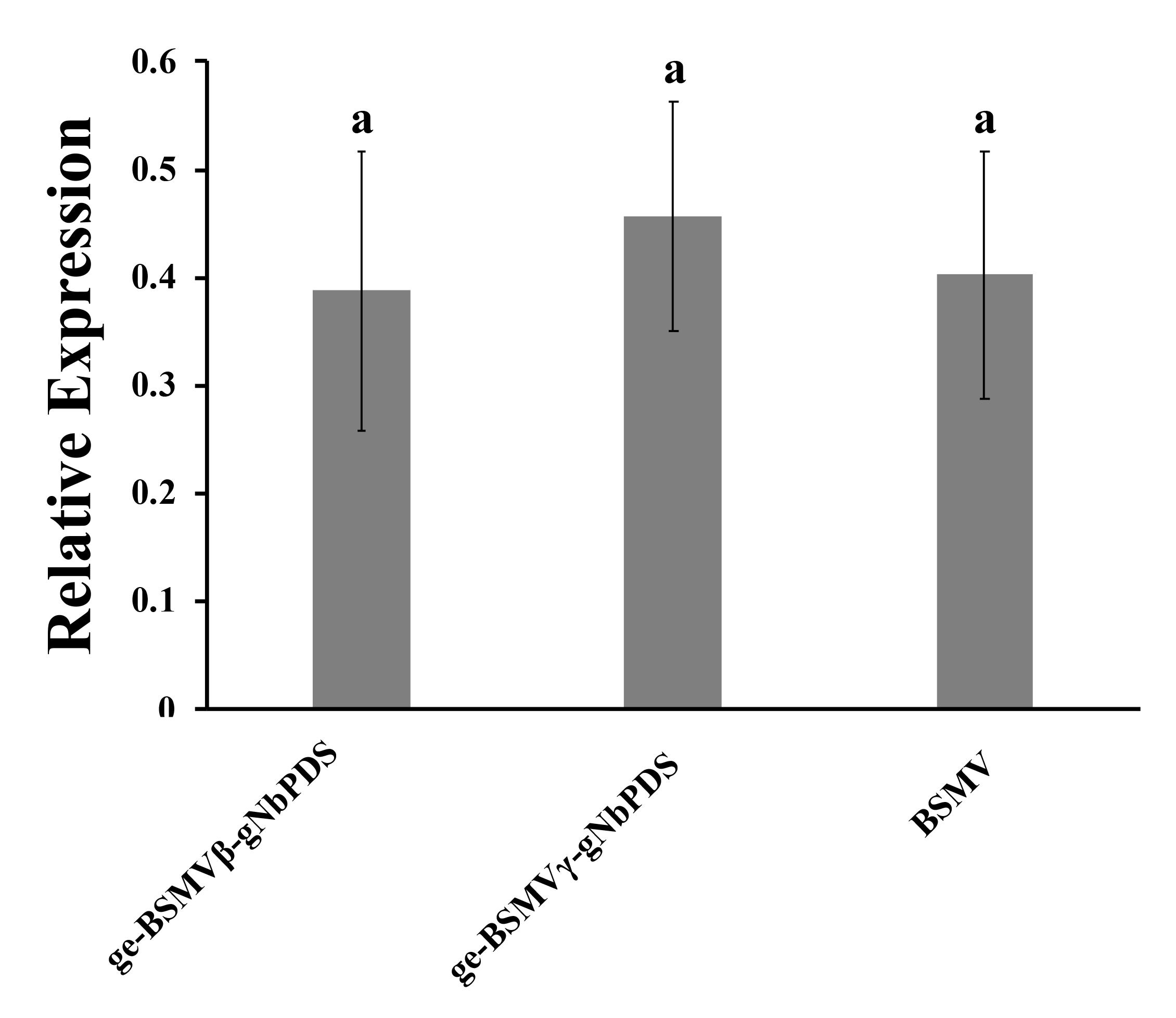

Supplement: Supplementary file 2 — Fig. S2 RT‐qPCR analysis of NbPDS mRNA level in ge‐BSMV infected N. benthamiana leaves. [file MPP-20-1463-s002.tif]

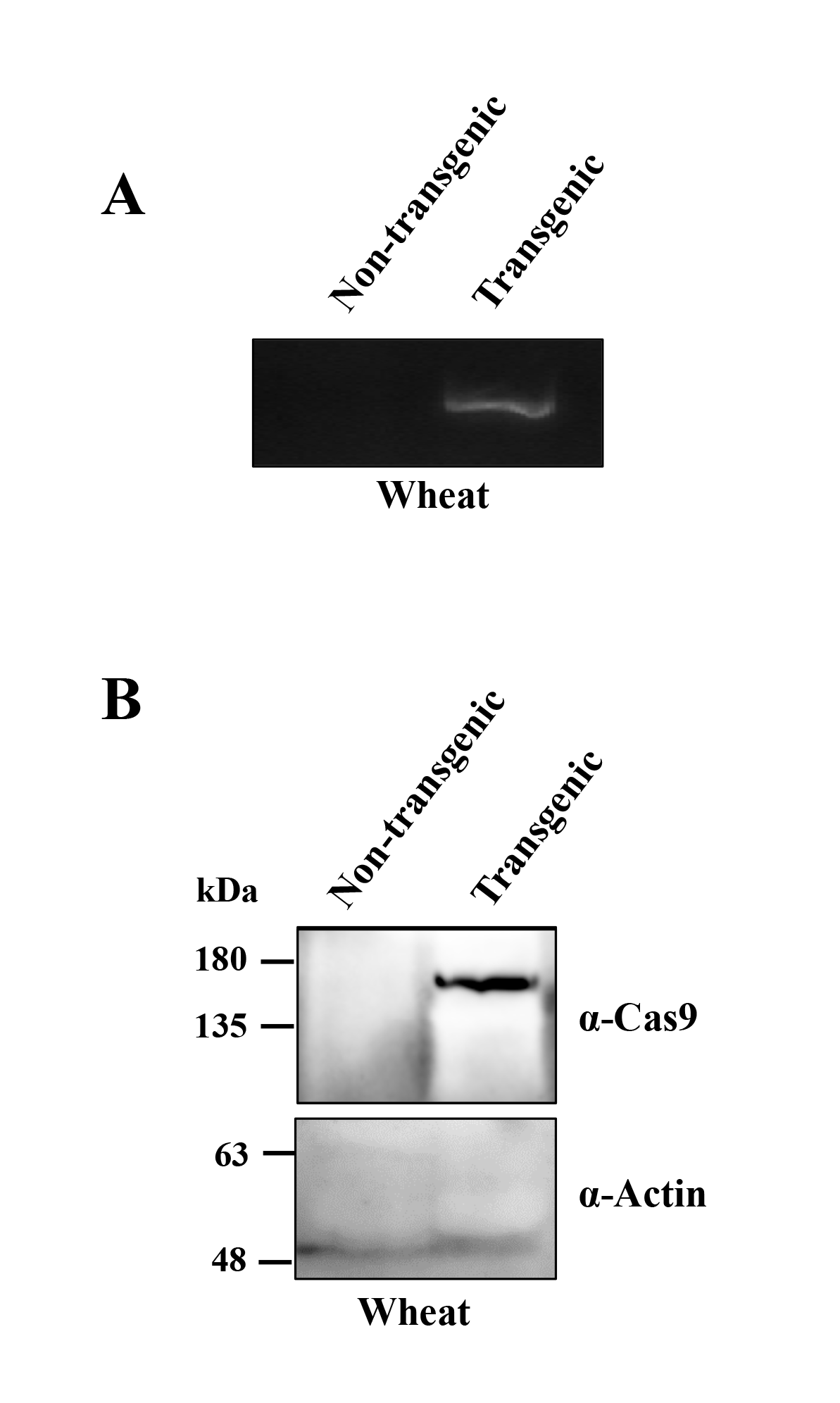

Supplement: Supplementary file 3 — Fig. S3 Genomic PCR (A) and western blot analysis (B) of Cas9‐transgenic wheat. For western blot analysis, the Cas9‐specific monoclonal antibody (Cat No.#14697, Cell Signaling Technology, Massachusetts, USA) was used at a 1:1000 dilution. [file MPP-20-1463-s003.tif]

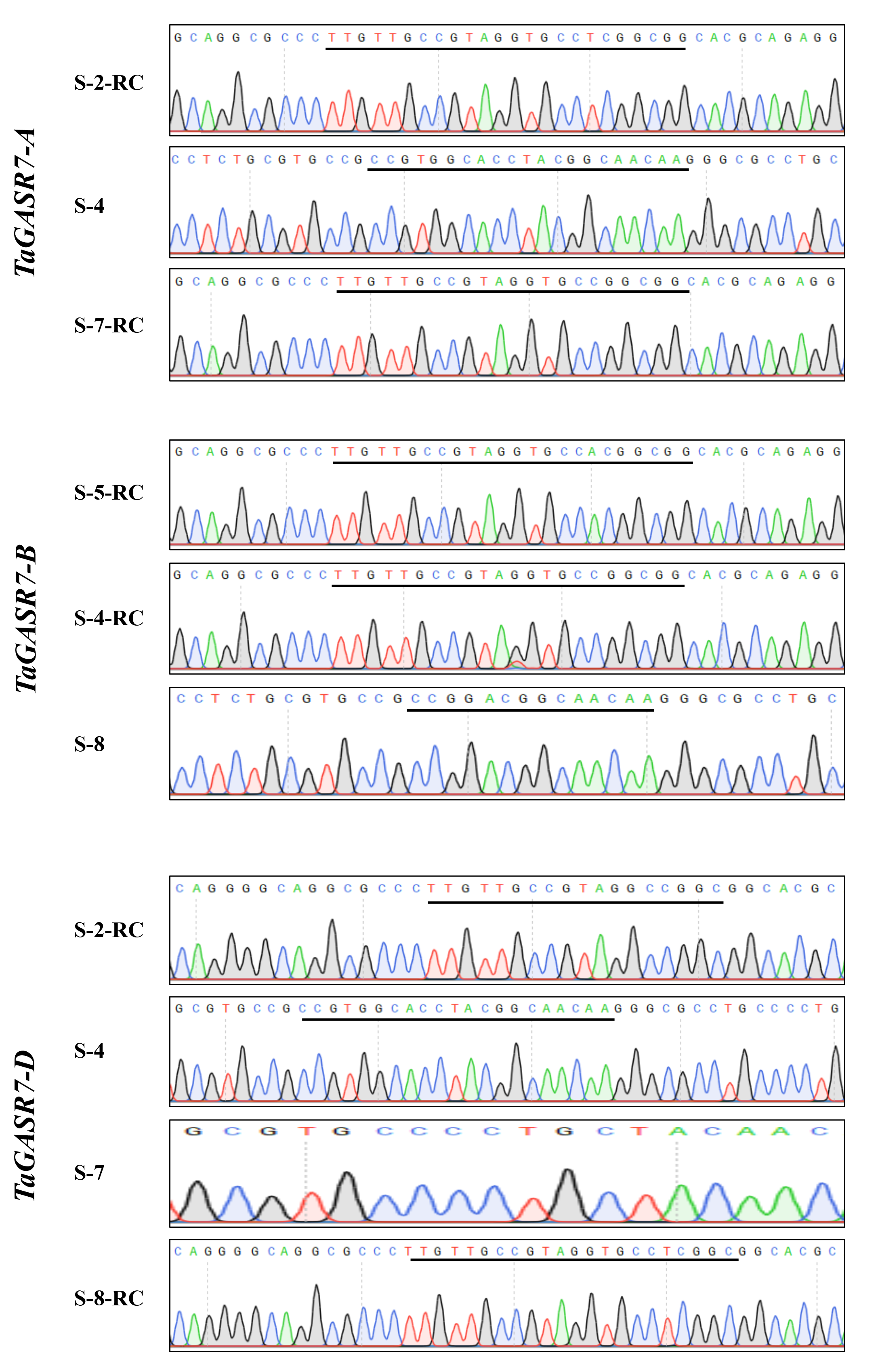

Supplement: Supplementary file 4 — Fig. S4 Representatives of Sanger sequencing chromatograms of indels from systemically infected wheat leaves. Black lines under the sequence indicates the target site. ‐RC, reverse complement sequence. [file MPP-20-1463-s004.tif]

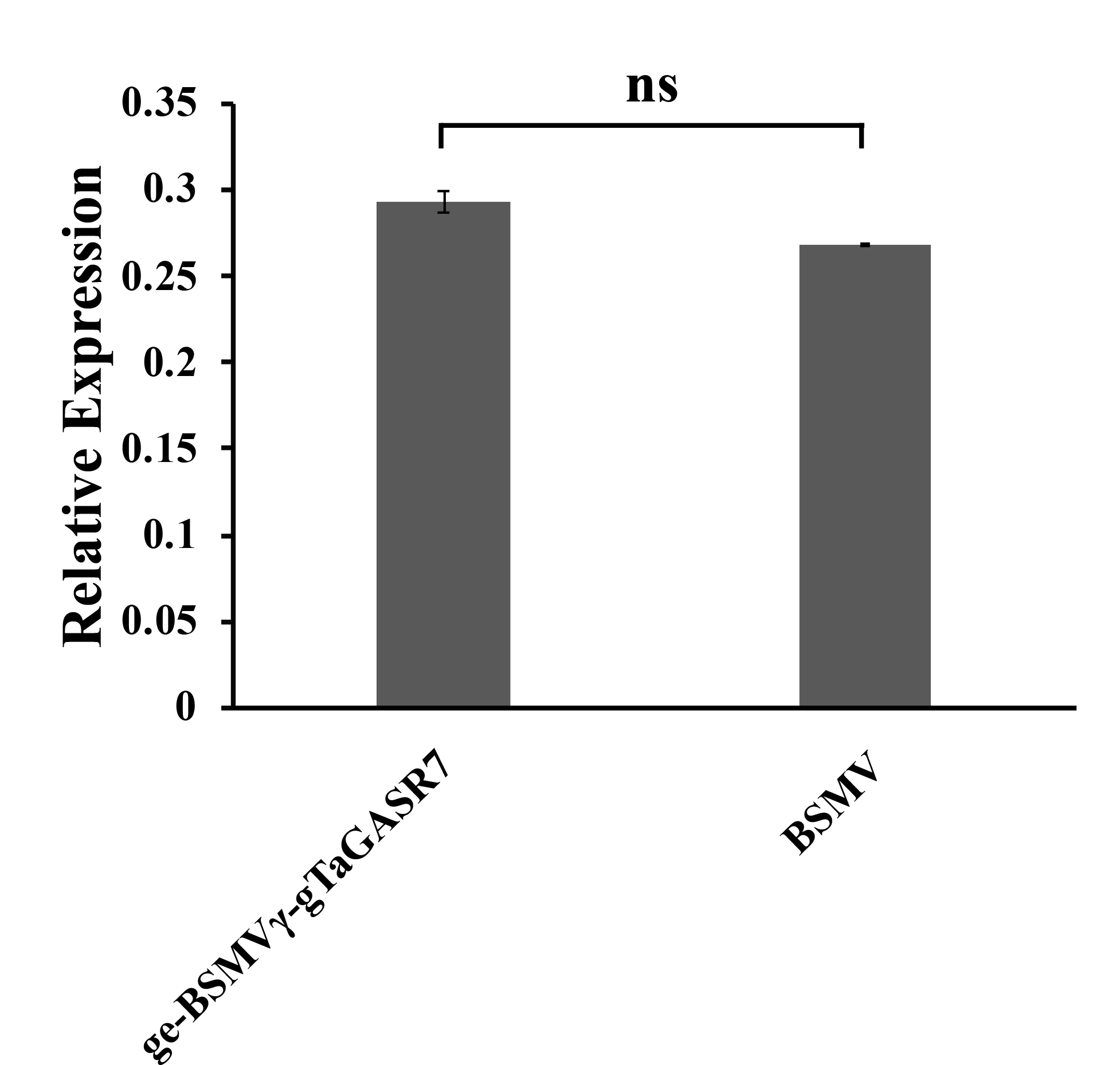

Supplement: Supplementary file 5 — Fig. S5 RT‐qPCR analysis of TaGASR7 mRNA levels in ge‐BSMV infected wheat leaves. Data are represented as means ± SD (Student’s t‐test; ns, not significant; n = 3). [file MPP-20-1463-s005.tif]

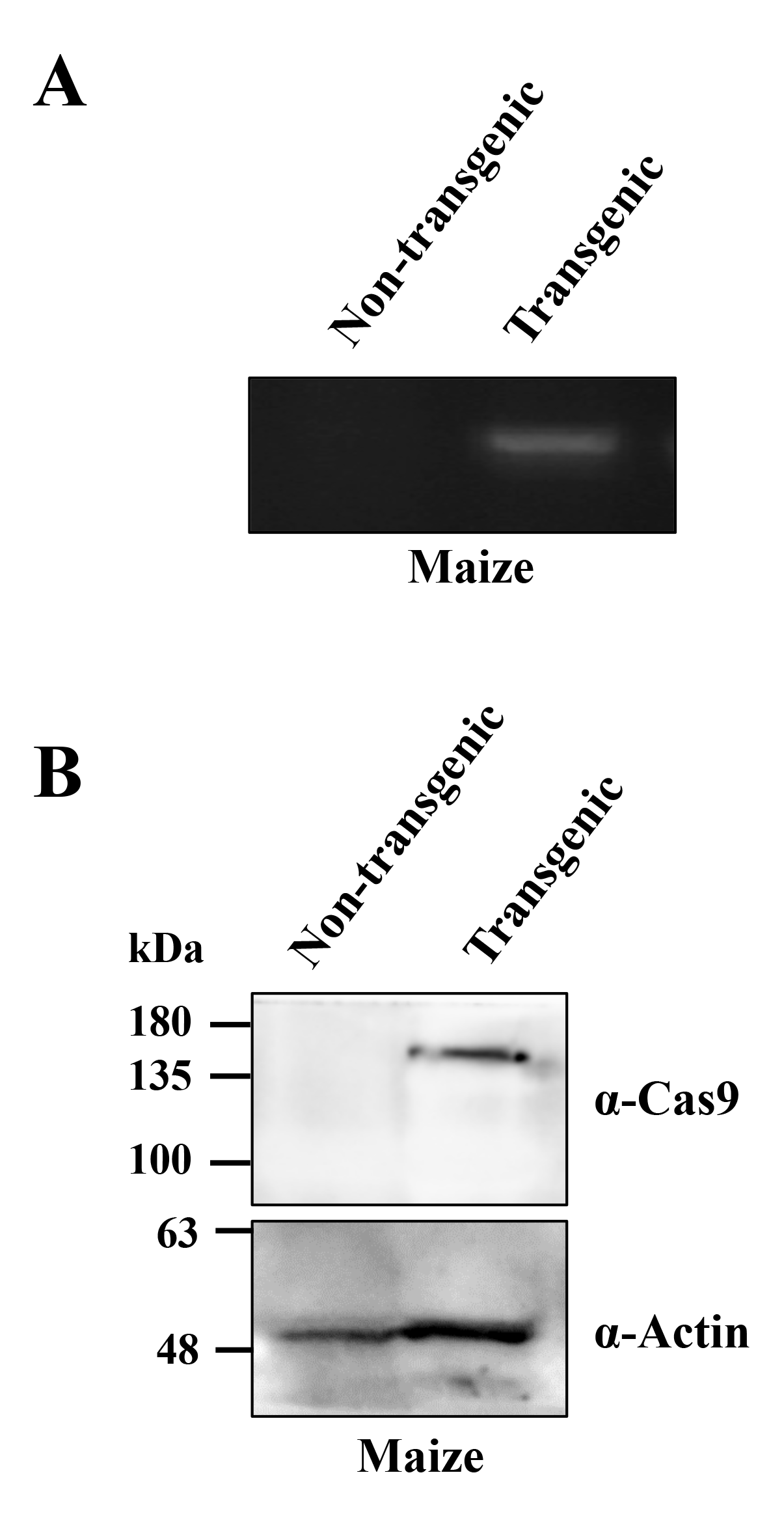

Supplement: Supplementary file 6 — Fig. S6 Genomic PCR (A) and western blot analysis (B) of the Cas9‐transgenic maize. For western blot analysis, Cas9 monoclonal antibody (Cat No.#14697, Cell Signaling Technology) was used with 1:1000 dilution. [file MPP-20-1463-s006.tif]

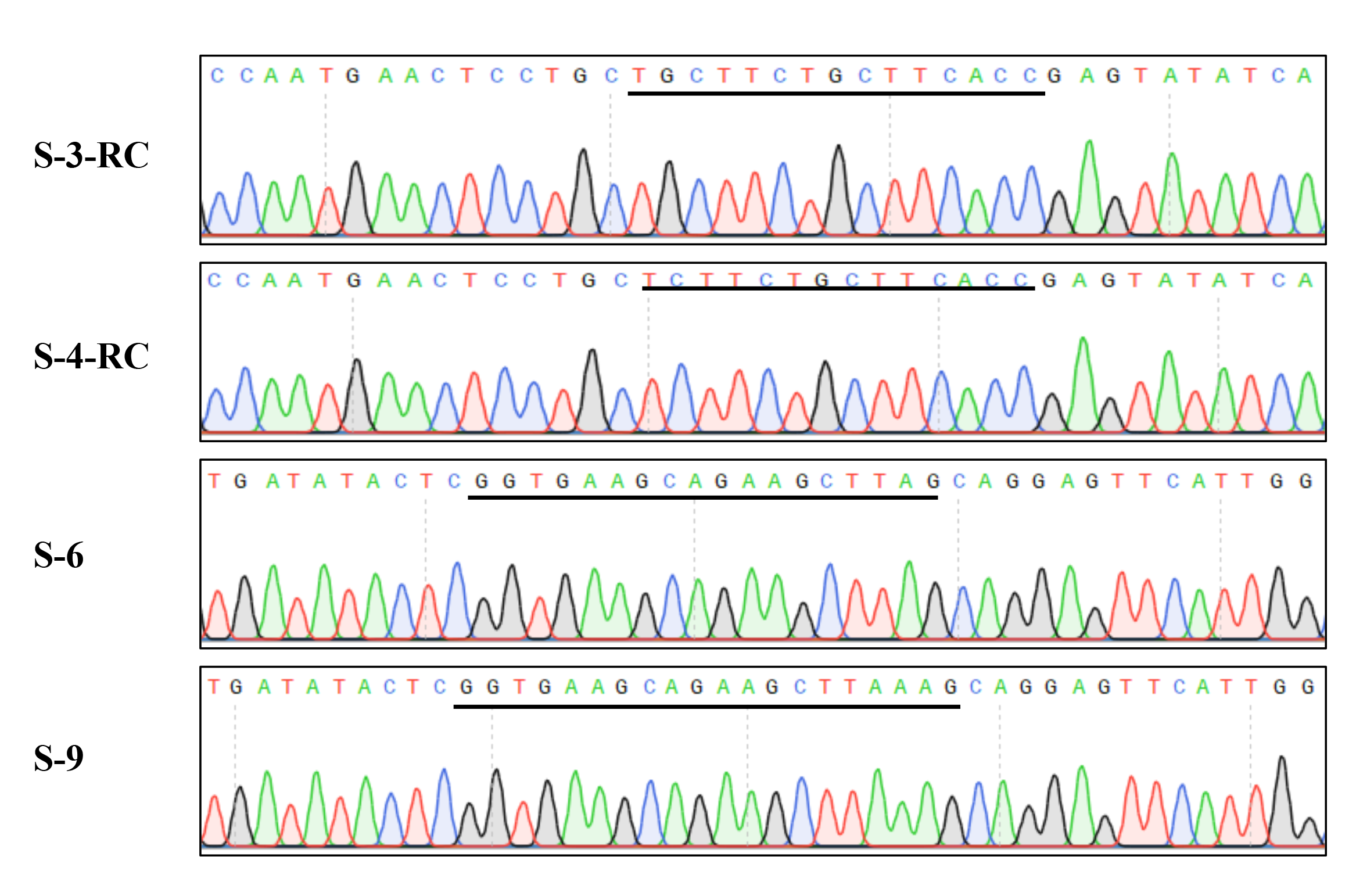

Supplement: Supplementary file 7 — Fig. S7 Representative Sanger sequencing chromatograms showing indels from systemically infected maize leaves. Black lines under the sequence indicates the target site. ‐RC, reverse complement sequence. [file MPP-20-1463-s007.tif]

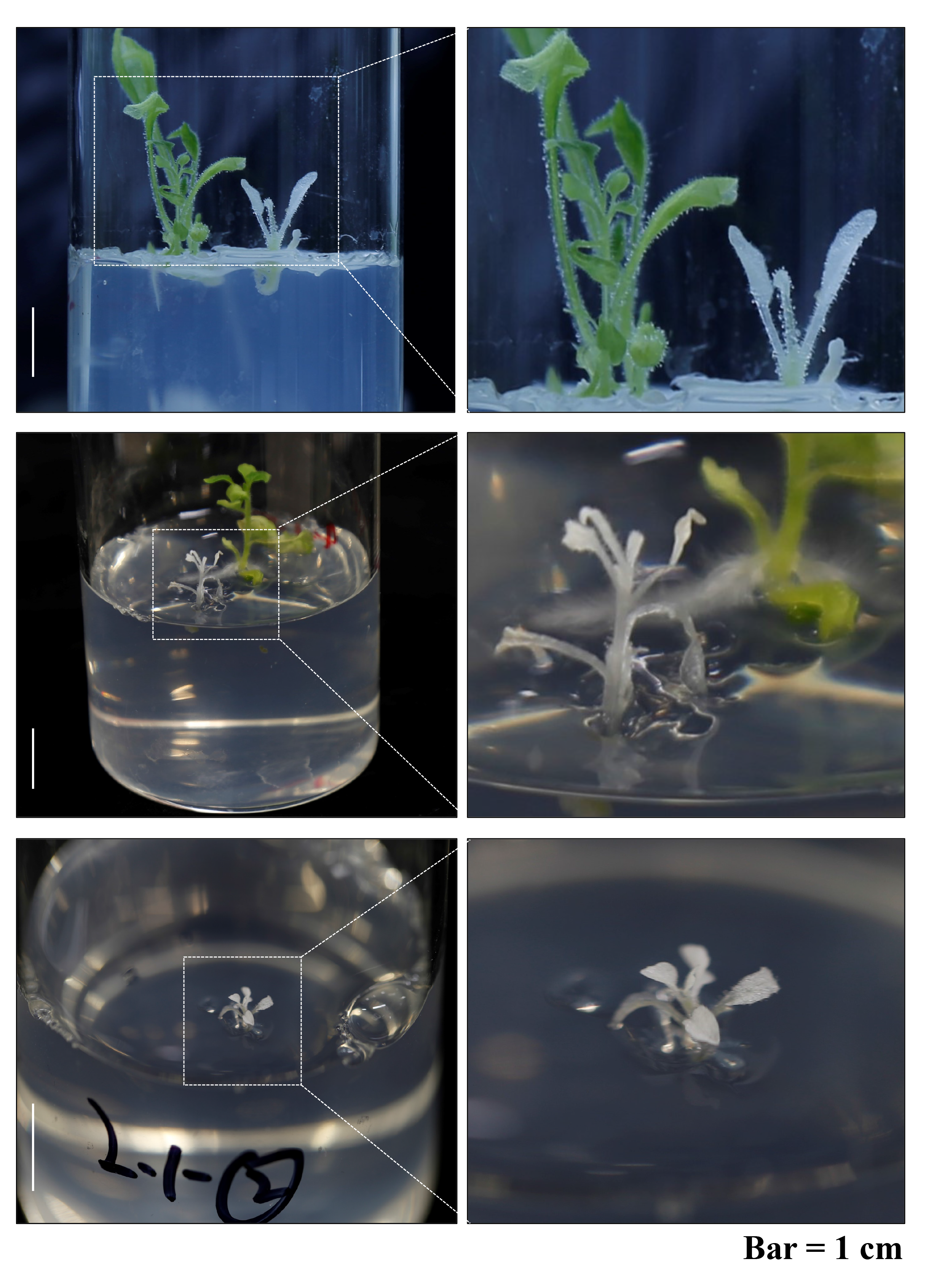

Supplement: Supplementary file 8 — Fig. S8 Regeneration of Nicotiana benthamiana leaf segments containing the edited NbPDS gene. ge‐BMVSγ‐gNbPDS‐infected systemically infected leaves of N. benthamiana were harvested at 30 days post‐inoculation (dpi), surface sterilized with 2.0–2.5% sodium hypochlorite, rinsed three times with sterile water, cut into c.2 cm² pieces and placed on medium plates (MS medium, 30.0 g/L sucrose, 1.0 mg/L zeatin, 2.0 mg/L kinetin, 1.0 mg/L indole‐3‐acetic acid, 350.0 mg/L carbenicillin, 4.0 g/L phytagel, pH 5.8) for differentiation under controlled conditions (23 °C, 16 h photoperiod). After 4–6 weeks, shoots with bleaching phenotypes were excised, transferred to rooting medium plates (1/3× MS medium, 7.0 g/L glucose, 3.0 g/L sucrose, 100.0 mg/L carbenicillin, 4.0 g/L phytagel, pH 5.8) and incubated at 23 °C. Representative N. benthamiana shoots regenerated from the leaf segments were photographed. Boxed regions in the left panels were magnified to show the regenerated albino plants. [file MPP-20-1463-s008.tif]
