# Supplementary material for: A barley stripe mosaic virus‐based guide RNA delivery system for targeted mutagenesis in wheat and maize
Source: Mol Plant Pathol. 2019 Jul 5;20(10):1463–74. doi: 10.1111/mpp.12849 (PMC6792137; doi:10.1111/mpp.12849)
Supplement: Supplementary file 9 — Table S1 Cas9 targets selected in this study. [file MPP-20-1463-s009.docx]

**Supplemental Table S1. Cas9 targets selected in this study.**

| **Target gene** | **Target site^a^** | **Restriction enzymes** | |
| --- | --- | --- | --- |
| *NbPDS* | TTTGGTAGTAGCGACTCCATGGG | | *Nco*I |
| *mGFP5* | GATACCCAGATCATATGAAGCGG | | *Nde*I |
| *TaGASR7* | TTGTTGCCGTAGGTGCCCGGCGG | | *Bcn*I |
| *ZmTMS5* | GGTGAAGCAGAAGCTTAAGCAGG | | *Afl*II |

^a^ Underlined letters indicate the restriction enzyme sites used in PCR/RC assay, letters in red indicate the PAM motif.
