# Supplementary material for: A barley stripe mosaic virus‐based guide RNA delivery system for targeted mutagenesis in wheat and maize
Source: Mol Plant Pathol. 2019 Jul 5;20(10):1463–74. doi: 10.1111/mpp.12849 (PMC6792137; doi:10.1111/mpp.12849)
Supplement: Supplementary file 10 — Table S2 Primers used in this study. [file MPP-20-1463-s010.docx]

**Supplemental Table S2. Primers used in this study.**

| **Primer** | **Primer sequence (5'-3') ^a^** | **Purpose** |
| --- | --- | --- |
| CP 74-393-F | GCCACAACTAACCCATCTCC | Reverse PCR to delete the nt 74-393 of BSMV CP ORF. |
| CP 74-393-R | CCACAACTTGTGTATCCCATTG |  |
| sgγ192-F | *GGGATACACAAGTTGTGG*GGTGCTTGATGCTTTGGATAAGGC | Amplification of the 192 bp sgγ promoter from RNAγ. |
| sgγ192-R | *CTTGCTATTTCTAGCTCTAAAAC*ACATCAGGACCTAGAGTTCACC |  |
| β-PDS-F1 | GACTCCATGTTTTAGAGCTAGAAATAGCAAG | Reverse PCR to insert the spacer to RNAβ-gNbPDS |
| β-PDS-R1 | GCTACTACCAAAaCATCAGGACCTAGAGTTC |  |
| TaGASR7-F | ctaTTGTTGCCGTAGGTGCCCGG | Construction of pCB301-ge-BSMVγ-TaGASR7 |
| TaGASR7-R | aacCCGGGCACCTACGGCAACAA |  |
| ZmTMS5-T2-F | ctaGGTGAAGCAGAAGCTTAAGC | Construction of pT7-ge-BSMVγ-gZmTMS5. |
| ZmTMS5-T2-R | aacGCTTAAGCTTCTGCTTCACC |  |
| ZmCas9-det-F | GCCAAGGGCTACAAGGAGG | Analysis of the Cas9-transgenic maize |
| ZmCas9-det-R | GATGGACTGGTGGATGAGGG |  |
| TaCas9-det-F | CGACCTCGACAATCTCCTCG | Analysis of the Cas9-transgenic wheat |
| TaCas9-det-R | GTAGTACGGGATGCGGAAGG |  |
| NbCas9-det-F | ACTCTGTTGGATGGGCTGTG | Analysis of the Cas9-transgenic *N.* *benthamiana*. |
| NbCas9-det-R | AGACCGAGAGAGAGTGCGAT |  |
| PDS-F | GCTTTGCTTGAGAAAAGCTCTC | Amplification of a 544 bp or a 743 bp DNA fragment flanking *NbPDS* target |
| NbPDS544-R | CCTTTAAAGGATTAAAGTCCTTTGTCA |  |
| NbPDS743-R | CCAATGGTTTAGTTGGGCGTG |  |
| TMS–994–F | TCAAGAGACTTGCGTCATCTTCCC | Amplification of a 994 bp DNA fragment flanking *ZmTMS5* target |
| TMS–994–R | GCATGCTCAACTGAAATTGAGTCGTC |  |
| TaGASR7-ABD-F | CCTTCATCCTTCAGCCATGCAT | Amplification of DNA fragments flanking *TaGASR7* target from wheat A, B, D genome |
| TaGASR7-A1-R | CCACTAAATGCCTATCACATACG |  |
| TaGASR7-B1-R | AGGGCAATTCACATGCCACTGAT |  |
| TaGASR7-D1-R | CCTCCATTTTTCCACATCTTAGTCC |  |
| BS11 | CACAAAATTAGGTAGAACTGATGTGAGAGATGT | Analysis of BSMV infection in systemic leaves. |
| BS32 | TGGTCTTCCCTTGGGGGACCGAA |  |
| pCB301-BSα-F | *CCTCTATATAAGGAAGTTCATTTCATTTGGAGAGG*GTATGTAAGTTGCCTTTGGGTG | Construction of the pCB301-BSMVα, pCB301-BSMVβ, and pCB301-BSMVγ. |
| pCB301-BSβ-F | *TATAAGGAAGTTCATTTCATTTGGAGAGG*GTAAAAGAAAAGGAACAACCCTGTTGTTGT |  |
| pCB301-BSγ-F | *CCTCTATATAAGGAAGTTCATTTCATTTGGAGAGG*GTATAGCTTGAGCATTACCG |  |
| pCB301-BS-R | *GGTGGAGATGCCATGCCGACCCGGG*TGGTCTTCCCTTGGGGGAC |  |
| pCB301-BSγ-MCS-F | ACTAGTGGGCCCTAAAAAAAAAAAAAAATGTTTGATC | Construction of pCB301-BSMVγ-MCS. |
| pCB301-BSγ-MCS-R | ACGCGTCCATGGCTTAGAAACGGAAGAAGAATCATCAC |  |
| Fb1 | *TGATTCTTCTTCCGTTTCTAAG*TAATTTGGTAGTAGCGACTCCATGTTTT | Construction of pCB301-BSMVγ-gNbPDS-mGFP5. |
| F1 | TTTGGTAGTAGCGACTCCATGTTTTAGAGCTAGAAATAGCAAGTTAAAATAAGG |  |
| R2 | CTTCATATGATCTGGGTATCAAAAAAAGCACCGACTCGGTGCC |  |
| Rb2 | *AACTTGCTATTTCTAGCTCTAA*AACCTTCATATGATCTGGGTATCAAAAAAAG |  |
| qNbPDS-F | GGTAACGGCCAAACCACCAC | RT-qPCR analysis of *NbPDS* mRNA accumulation. |
| qNbPDS-R | GTGTTTCACGTTGTGGACTATGC |  |
| PP2A-realtime-F | GACCCTGATGTTGATGTTCGCT | Reference gene for RT-qPCR analysis of *NbPDS* mRNA accumulation. |
| PP2A-realtime-R | GAGGGATTTGAAGAGAGATTTC |  |
| qGASR-F1 | GGGACGCAGTACAAGAAGGC | RT-qPCR analysis of *TaGASR7* mRNA accumulation. |
| qGASR-R1 | TTCCAGTTGTTGTAGCAGGGG |  |
| TaEF1α-F | CAAGGCTGCCATCAAGAAGAAA | Reference gene for RT-qPCR analysis of *TaGASR7* mRNA accumulation. |
| TaEF1α-R | AACAACGCCCAGGAATAACACT |  |

^a^ Underlined letters indicate restriction enzyme sites, lowercase letters indicate overhang sequence used for oligos annealing and ligation, italicized letters indicate sequence used for seamless assembly cloning.
