# Supplementary material for: A barley stripe mosaic virus‐based guide RNA delivery system for targeted mutagenesis in wheat and maize
Source: Mol Plant Pathol. 2019 Jul 5;20(10):1463–74. doi: 10.1111/mpp.12849 (PMC6792137; doi:10.1111/mpp.12849)
Supplement: Supplementary file 11 — File S1 Sequences of RNAβ‐gNbPDS, RNAγ‐gNbPDS, RNAγ‐gTaGASR7, RNAγ‐gZmTMS5, RNAγ‐gNbPDS‐mGFP5, RNAγ‐SmR and pCB301‐BSMVγ‐MCS. Sequences in green indicate the sgRNAγ promoter, whereas sequences in red, blue and purple represent the gRNA spacer, the scaffold and the SmR gene, respectively. The underlined sequences indicate the SapI recognition sites or the multiple cloning sites. [file MPP-20-1463-s011.docx]

**Supplemental Sequences and Maps**

**Sequence of RNAβ-gNbPDS.** Sequence in green indicates the sgγ promoter, sequence in red indicates the sgRNA spacer, and sequence in blue indicates the scaffold.

GTAAAAGAAAAGGAACAACCCTGTTGTTGTTCGACGCTATACTAAATATATATTATCTTATTAGTGCATTTCTTTTACCACTTCACAGTATGCCGAACGTTTCTTTGACTGCTAAGGGTGGAGGACACTACAACGAGGATCAATGGGATACACAAGTTGTGGGGTGCTTGATGCTTTGGATAAGGCTTTAGTGGATAGATATCGATCCCCTTATAGTGCTATTTCTGCTTTGGTTTCTTTATGTTATCATATCTTTGACTTTAATAAGTTTAAGTTGCTGTTTAATTGTGAAGGGAAATTTGTGGATAAGAAGCTGAGAAAAGACTTCGAGTGGTGAACTCTAGGTCCTGATGtTTTGGTAGTAGCGACTCCATGTTTTAGAGCTAGAAATAGCAAGTTAAAATAAGGCTAGTCCGTTATCAACTTGAAAAAGTGGCACCGAGTCGGTGC**TTTTTTT**GCCACAACTAACCCATCTCCCCCGGCGCAGGCACCGTCGGAGAATCTTACTCTTCGTGATGTTCAACCGTTAAAGGATAGTGCGTTGCATTATCAATACGTGTTGATTGACCTACAGAGTGCGAGACTCCCAGTGTATACCAGGAAGACTTTCGAACGTGAACTCGCTTTGGAATGGATCATTCCAGATGCCGAGGAAGCGTGACCTGCTGTTGAAGCGGTAAAAGGATGTACATATGTATCTTATTTATTTTGTTTATCTATTTTCTTTTACTTTTAGTTTTTGCTTTTTACGCGTTAACTAGATGTATTGACTTTAGCCATGGACATGACGAAAACTGTTGAGGAAAAGAAAACAAATGGAACTGATTCAGTGAAAGGTGTTTTTGAAAACTCGACGATTCCCAAAGTTCCGACTGGACAGGAAATGGGTGGTGACGATTCTTCTACTTCTAAATTAAAGGAAACTCTAAAAGTTGCCGATCAGACTCCATTGTCCGTTGACAACGGTGCCAAATCCAAATTGGATTCTTCTGATAGACAAGTTCCTGGTCCTAAGTTGGCAACAACTGTGGAAAAGGAACCTGAGTTGAAACCCAACGTTAAGAAGTCCAAGAAGAAAAGAATCCAAAAACCTGCTCAACCGAGTAGGCCCAATGACCTTAAAGGCGGGACTAAGGGATCATCTCAAGTGGGTGAAAATGTGAGTGAGAACTATACTGGGATTTCTAAGGAAGCAGCTAAGCAAAAGCAGAAGACACCCAAGTCTGTGAAAATGCAAAGCAATCTGGCCGATAAGTTCAAAGCGAATGATACTCGTAGATCGGAATTAATTAACAAGTTTCAGCAATTTGTGCATGAAACCTGTCTTAAATCTGATTTTGAGTACACTGGTCGACAGTATTTCAGAGCTAGATCAAATTTCTTTGAAATGATTAAGCTCGCATCCTTGTATGACAAACATCTAAAGGAATGTATGGCGCGAGCCTGCACCCTAGAACGAGAACGATTGAAGCGTAAGTTACTCCTAGTACGAGCTTTGAAACCAGCAGTTGACTTCCTTACGGGAATCATCTCTGGAGTTCCTGGCTCAGGAAAATCAACCATTGTGCGTACTTTGCTCAAAGGTGAATTTCCGGCTGTTTGTGCTTTGGCCAATCCTGCCTTAATGAACGACTATTCTGGTATTGAAGGCGTTTACGGGTTAGATGACCTGTTGCTTTCTGCAGTTCCGATAACGTCTGATTTATTGATCATAGATGAATATACACTTGCTGAGAGCGCGGAAATCCTGTTGTTACAACGAAGACTCAGAGCCTCTATGGTGTTGTTAGTCGGGGATGTAGCTCAAGGAAAAGCCACCACTGCTTCCAGTATTGAGTATTTAACTCTGCCGGTGATCTACAGATCAGAGACGACTTATCGTTTGGGACAAGAGACTGCTTCGCTTTGCAGCAAGCAGGGTAACAGAATGGTTTCAAAGGGTGGAAGGGACACAGTGATCATTACTGATTACGATGGCGAAACAGATGGAACGGAGAAAAATATCGCTTTTACTGTCGATACAGTTCGAGATGTGAAAGATTGCGGGTACGATTGTGCCCTGGCAATTGATGTGCAAGGGAAAGAATTCGATTCAGTGACTTTATTCCTAAGGAACGAAGACCGGAAAGCTTTAGCAGATAAGCATTTGCGTTTAGTCGCTTTGAGCAGACATAAGTCGAAGTTAATCATCAGGGCCGACGCGGAAATTCGTCAAGCATTCCTGACAGGTGATATTGACTTGAGCTCTAAGGCGAGTAACTCTCATCGTTATTCTGCAAAACCGGATGAAGACCACAGTTGGTTCAAGGCCAAATAAGTATTGGCCAATTGTCGCCGGAATCGGTGTCGTTGGATTGTTTGCGTATTTGATCTTTTCAAATCAAAAACATTCTACGGAATCCGGCGATAATATTCACAAATTCGCCAACGGAGGTAGTTACAGGGACGGGTCAAAGAGTATAAGTTATAATCGTAATCATCCTTTTGCCTATGGCAATGCCTCATCCCCTGGAATGTTGTTGCCCGCAATGCTTACCATCATCGGAATCATTTCCTATTTATGGCGAACAAGAGATTCCGTGCTCGGAGACTCAGGCGGAAACAATTCCTGCGGAGAAGACTGTCAGGGCGAATGTCTTAACGGACATTCTCGACGATCATTACTATGCGATATTGGCTAGTCTTTTTATCATTGCTCTATGGTTATTGTATATATATCTAAGCAGTATACCTACGGAGACTGGTCCCTACTTCTATCAAGATCTGAACTCTGTGAAGATCTATGGAATAGGGGCTACGAATCCAGAAGTTATTGCGGCCATCCACCATTGGCAGAAGTACCCTTTTGGGGAATCTCCGATGTGGGGAGGTTTAGTCAGTGTTTTGAGCGTTCTTCTTAAACCGCTGACGTTAGTTTTTGCGTTAAGCTTTTTTCTCTTACTTTCTTCAAAAAGGTAAAAAAAAAAAAAAAAAAAATGTTTGATCAGATCATTCAAATCTGATGGTGCCCATCAACCATATGATGGGAGTGTTTGCAAGTCCACTATAATCGAACTTGAAAACGATGCCTGAATTGGAAACCATGAATCTTAACGGATTCTGGAGAGAAAATTTAGGAATTGGTATGTAAGCTACAACTTCCGGTAGCTGCGTCACACTTTAAGAGTGTGCATACTGAGCCGAAGCTCAGCTTCGGTCCCCCAAGGGAAGACCA

**Sequence of RNAγ-gNbPDS.** Sequence in red indicates the sgRNA spacer, sequence in blue indicates the scaffold.

GTATAGCTTGAGCATTACCGTCGTGTAATTGCAACACTTGGCTTGCCAAATAACGCTAAAGCGTTCACGAAACAAACAACACTTCGGCATGGATGTTGTGAAGAAATTCGCCGTCATGTCAGTGACTGTAGTAGCAGGTCCCGTCCTTACGCTTTCATCACCTGTGGTGGTGACGTTTGGAACAGGCTTAATTGCCGTATCTTTGGTGAAACGGTTGCTACAGGAACAACCCCGTGTAATTGCTCACGATCACGAACATTACCCAGGTGGTTCTGAGAGCAGTTCTAGCTCTTGTGCTACCGCGCCTATTTTACGTAATCTTTCGCGAGATCAGTGCGATTCAGAGAATATTGGATGCAGTTCTAGCGCCTGTTCTCCGTCTGAAATTGTGAAAGTTACAAGGCAGGTAGTGGGAGTTGAACGTGGTCTTTACCGGGACATTTTTCAGGACAACGAAATCCCATCAGTCATGGAAGAGAAACTGCAGAAACTCCTTTACTCTGAGGGTGAGAAGATTCGAAGACGTTGCCAATTTGAAGCATCAACGATGCACTCACGCAAAGTAAAGGTTCCGGAGGTAGGTACTATCCCAGATATCCAAACTTGGTTCGATGCTACGTTTCCTGGTAACTCCGTTAGGTTTTCTGATTTCGACGGTTATACTGTTGCTACGGAGGACATTAACATGGATGTTCAGGATTGTAGACTTAAGTTCGGGAAGACTTTTCGACCTTATGAATTTAAGGAATCACTGAAACCAGTACTGAGGACAGCAATGCCAGAAAAACGACAGGGTAGTTTGATTGAAAGTGTGCTGGCCTTTCGTAAAAGAAATTTGGCTGCGCCCAGATTACAAGGAGCTTTGAATGAATGGCACACAATTGAGAATGTGCTAACGAAGGCGTTAAAGGTATTCTTCTTTGAAGATTTAATTGATCGAACGGATCACTGCACTTACGAGTCAGCGCTCAGATGGTGGGATAAACAATCAGTGACAGCTCGAGCGCAGCTCGTGGCGGATCAGCGGAGGTTATGTGATGTTGACTTCACGACTTATAACTTCATGATAAAAAATGATGTAAAGCCGAAGTTAGATCTAACACCTCAAGTTGAATATGCAGCTTTGCAGACTGTTGTATATCCTGATAAGATAGTCAATGCTTTCTTTGGTCCGATCATAAAGGAGATTAATGAACGGATCATCAGAGCGCTTAGACCTCATGTGGTCTTTAATTCTCGTATGACTGCTGATGAACTGAATGAAACAGCTGCCTTTTTGACACCTCATAAGTACAGAGCCTTAGAGATTGATTTTTCAAAATTTGATAAATCAAAGACTGGGCTTCATATCAAAGCTGTCATTGGACTCTATAAGCTCTTTGGCCTAGATGGCCTGTTAAAAGTGCTCTGGGAAAAATCGCAATATCAGACTTACGTGAAAGATAGAAACTTCGGTCTCGAGGCATATCTATTGTATCAGCAAAAGTCAGGAAATTGTGACACTTACGGTTCGAACACCTGGTCTGCCGCCTTGGCGTTGTTAGATTGTCTTCCTTTGGAAGATGCACATTTCTGTGTATTTGGTGGTGATGATTCATTGATATTGTTTGATCAGGGATACATAATTTCCGACCCATGCCGGCAACTTGCCGGTACTTGGAATCTTGAATGTAAAGTGTTCGACTTCAAGTACCCCGCATTTTGTGGTAAATTTCTGCTGTGCATAGATGGAAAATATCAATTTGTTCCAGATGCGGCAAAATTTATCACAAAATTAGGTAGAACTGATGTGAGAGATGTAGAAGTTTTGAGTGAGATTTATATCTCTATCAATGACAATTACAAATCTTACAAAGACTTTAAGGTGCTTGATGCTTTGGATAAGGCTTTAGTGGATAGATATCGATCCCCTTATAGTGCTATTTCTGCTTTGGTTTCTTTATGTTATCATATCTTTGACTTTAATAAGTTTAAGTTGCTGTTTAATTGTGAAGGGAAATTTGTGGATAAGAAGCTGAGAAAAGACTTCGAGTGGTGAACTCTAGGTCCTGATGTTTAAATCTACTGTATTTACCTTCGCATGATGGCTACTTTCTCTTGTGTGTGTTGTGGTACCTTAACTACAAGTACTTACTGTGGTAAGAGATGTGAGCGAAAGCATGTATATTCTGAAACAAGAAATAAGAGATTGGAACTTTACAAGAAGTATCTATTGGAACCGCAAAAATGCGCCCTGAATGGAATCGTTGGACACAGTTGTGGAATGCCATGCTCCATTGCGGAAGAGGCTTGTGATCAACTGCCAATCGTGAGTAGGTTCTGTGGCCAAAAGCATGCGGATCTGTATGATTCACTTCTGAAACGTTCTGAACAGGAGTTACTTCTTGAATTTCTCCAGAAGAAGATGCAGGAGCTGAAACTTTCTCATATCGTAAAAATGGCTAAGCTTGAAAGTGAGGTTAACGCAATACGTAAGTCCGTAGCTTCTTCTTTTGAAGATTCTGTTGGATGTGATGATTCTTCTTCCGTTTCTAAGTAACCATGGTTTGGTAGTAGCGACTCCATGTTTTAGAGCTAGAAATAGCAAGTTAAAATAAGGCTAGTCCGTTATCAACTTGAAAAAGTGGCACCGAGTCGGTGC**TTTTTTT**ACTAGTGGGCCCTAAAAAAAAAAAAAAATGTTTGATCAGATCATTCAAATCTGATGGTGCCCATCAACCATATGATGGGAGTGTTTGCAAGTCCACTATAATCGAACTTGAAAACGATGCCTGAATTGGAAACCATGAATCTTAACGGACTCTGGAGAGAAAATTTAGGAATTGGTATGTAAGCTACAACTTCCGGTAGCTGCGTCACACTTTAAGAGTGTGCATACTGAGCCGAAGCTCAGCTTCGGTCCCCCAAGGGAAGACCA

**Sequence of RNAγ-gTaGASR7.** Sequence in red indicates the sgRNA spacer, sequence in blue indicates the scaffold.

GTATAGCTTGAGCATTACCGTCGTGTAATTGCAACACTTGGCTTGCCAAATAACGCTAAAGCGTTCACGAAACAAACAACACTTCGGCATGGATGTTGTGAAGAAATTCGCCGTCATGTCAGTGACTGTAGTAGCAGGTCCCGTCCTTACGCTTTCATCACCTGTGGTGGTGACGTTTGGAACAGGCTTAATTGCCGTATCTTTGGTGAAACGGTTGCTACAGGAACAACCCCGTGTAATTGCTCACGATCACGAACATTACCCAGGTGGTTCTGAGAGCAGTTCTAGCTCTTGTGCTACCGCGCCTATTTTACGTAATCTTTCGCGAGATCAGTGCGATTCAGAGAATATTGGATGCAGTTCTAGCGCCTGTTCTCCGTCTGAAATTGTGAAAGTTACAAGGCAGGTAGTGGGAGTTGAACGTGGTCTTTACCGGGACATTTTTCAGGACAACGAAATCCCATCAGTCATGGAAGAGAAACTGCAGAAACTCCTTTACTCTGAGGGTGAGAAGATTCGAAGACGTTGCCAATTTGAAGCATCAACGATGCACTCACGCAAAGTAAAGGTTCCGGAGGTAGGTACTATCCCAGATATCCAAACTTGGTTCGATGCTACGTTTCCTGGTAACTCCGTTAGGTTTTCTGATTTCGACGGTTATACTGTTGCTACGGAGGACATTAACATGGATGTTCAGGATTGTAGACTTAAGTTCGGGAAGACTTTTCGACCTTATGAATTTAAGGAATCACTGAAACCAGTACTGAGGACAGCAATGCCAGAAAAACGACAGGGTAGTTTGATTGAAAGTGTGCTGGCCTTTCGTAAAAGAAATTTGGCTGCGCCCAGATTACAAGGAGCTTTGAATGAATGGCACACAATTGAGAATGTGCTAACGAAGGCGTTAAAGGTATTCTTCTTTGAAGATTTAATTGATCGAACGGATCACTGCACTTACGAGTCAGCGCTCAGATGGTGGGATAAACAATCAGTGACAGCTCGAGCGCAGCTCGTGGCGGATCAGCGGAGGTTATGTGATGTTGACTTCACGACTTATAACTTCATGATAAAAAATGATGTAAAGCCGAAGTTAGATCTAACACCTCAAGTTGAATATGCAGCTTTGCAGACTGTTGTATATCCTGATAAGATAGTCAATGCTTTCTTTGGTCCGATCATAAAGGAGATTAATGAACGGATCATCAGAGCGCTTAGACCTCATGTGGTCTTTAATTCTCGTATGACTGCTGATGAACTGAATGAAACAGCTGCCTTTTTGACACCTCATAAGTACAGAGCCTTAGAGATTGATTTTTCAAAATTTGATAAATCAAAGACTGGGCTTCATATCAAAGCTGTCATTGGACTCTATAAGCTCTTTGGCCTAGATGGCCTGTTAAAAGTGCTCTGGGAAAAATCGCAATATCAGACTTACGTGAAAGATAGAAACTTCGGTCTCGAGGCATATCTATTGTATCAGCAAAAGTCAGGAAATTGTGACACTTACGGTTCGAACACCTGGTCTGCCGCCTTGGCGTTGTTAGATTGTCTTCCTTTGGAAGATGCACATTTCTGTGTATTTGGTGGTGATGATTCATTGATATTGTTTGATCAGGGATACATAATTTCCGACCCATGCCGGCAACTTGCCGGTACTTGGAATCTTGAATGTAAAGTGTTCGACTTCAAGTACCCCGCATTTTGTGGTAAATTTCTGCTGTGCATAGATGGAAAATATCAATTTGTTCCAGATGCGGCAAAATTTATCACAAAATTAGGTAGAACTGATGTGAGAGATGTAGAAGTTTTGAGTGAGATTTATATCTCTATCAATGACAATTACAAATCTTACAAAGACTTTAAGGTGCTTGATGCTTTGGATAAGGCTTTAGTGGATAGATATCGATCCCCTTATAGTGCTATTTCTGCTTTGGTTTCTTTATGTTATCATATCTTTGACTTTAATAAGTTTAAGTTGCTGTTTAATTGTGAAGGGAAATTTGTGGATAAGAAGCTGAGAAAAGACTTCGAGTGGTGAACTCTAGGTCCTGATGTTTAAATCTACTGTATTTACCTTCGCATGATGGCTACTTTCTCTTGTGTGTGTTGTGGTACCTTAACTACAAGTACTTACTGTGGTAAGAGATGTGAGCGAAAGCATGTATATTCTGAAACAAGAAATAAGAGATTGGAACTTTACAAGAAGTATCTATTGGAACCGCAAAAATGCGCCCTGAATGGAATCGTTGGACACAGTTGTGGAATGCCATGCTCCATTGCGGAAGAGGCTTGTGATCAACTGCCAATCGTGAGTAGGTTCTGTGGCCAAAAGCATGCGGATCTGTATGATTCACTTCTGAAACGTTCTGAACAGGAGTTACTTCTTGAATTTCTCCAGAAGAAGATGCAGGAGCTGAAACTTTCTCATATCGTAAAAATGGCTAAGCTTGAAAGTGAGGTTAACGCAATACGTAAGTCCGTAGCTTCTTCTTTTGAAGATTCTGTTGGATGTGATGATTCTTCTTCCGTTTCTAAGTAactATTGTTGCCGTAGGTGCCCGGGTTTTAGAGCTAGAAATAGCAAGTTAAAATAAGGCTAGTCCGTTATCAACTTGAAAAAGTGGCACCGAGTCGGTGC**TTTTTTT**AAAAAAAAAAAAATGTTTGATCAGATCATTCAAATCTGATGGTGCCCATCAACCATATGATGGGAGTGTTTGCAAGTCCACTATAATCGAACTTGAAAACGATGCCTGAATTGGAAACCATGAATCTTAACGGACTCTGGAGAGAAAATTTAGGAATTGGTATGTAAGCTACAACTTCCGGTAGCTGCGTCACACTTTAAGAGTGTGCATACTGAGCCGAAGCTCAGCTTCGGTCCCCCAAGGGAAGACCA

**Sequence of RNAγ-gZmTMS5.** Sequence in red indicates the sgRNA spacer, sequence in blue indicates the scaffold.

gtatagcttgagcattaccgtcgtgtaattgcaacacttggcttgccaaataacgctaaagcgttcacgaaacaaacaacaattcggcatggatgttgtgaagaaattcgccgtcatgtcagtgactgtagtagcaggtcccgtccttacgctttcatcacctgtggtggtgacgtttggaacaggcttaattgccgcatctttggtgaaacggttgctacaggaacaaccccgtgtaattgctcacgatcacgaacattacccaggtggttctgagagcagttctagctcttgtgctaccgcgcctattttacgtaatctttcgcgagatcagtgcgattcagagaatattggatgcagttctagcgcctgttctccgtctgaaattgtgaaagttacaaggcaggtagtggaagttgaacgtggtctttaccgggacatttttcaggacaacgaaatcccatcagtcatggaagagaaactgcagaaactcctttactctgagggtgagaagattcgaagacgttgccaatttgaagcatcaacgatgcactcacgcaaagtaaaggttccggaggtaggtactatcccagatatccaaacttggttcgatgctacgtttcctggtaactccgttaggttttctgatttcgacggttatactgttgctacggaggacattaacatggatgttcaggattgtagacttaagttcgggaagacttttcgaccttatgaatttaaggaatcactgaaaccagtactgaggacagcaatgccagaaaagcgacagggtagtttgattgaaagtgtgctggcctttcgtaaaagaaatttggctgcgcccagattacaaggagctttgaatgaatggcacacaattgagaatgtgctgaagaaggcgttaaaggtattcttctttgaagatttaattgatcgaacggatcattgcacttacgagtcagcgctcagatggtgggataaacaatcagtgacagctcgagcgcagctcgtggcggaccagcggaggttatgtgatgttgacttcacgacttataacttcatgataaaaaatgatgtaaagccgaagttggatctaacacctcaagttgaatatgcagctttgcagactgttgtgtatcctgataagatagtcaatgctttctttggtccgatcataaaggagattaatgaacggatcatcagagcgcttagacctcatgtggtctttaattctcgtatgactgctgatgaactgaatgaaacagttgcctttttgacacctcacaagtacagagccttagagattgacttttcaaaatttgataaatcaaagactgggcttcatatcaaagctgtcattggactctataagctctttggtctagatggcctgttaaaagtactctgggaaaaatcgcaatatcagacttacgtgaaagatagaaacttcggtctcgaggcatatctattatatcagcaaaagtcaggaaattgtgacacttacggttcgaacacctggtctgccgccttggcgttgttagattgtcttcctttggaagatgcacatttctgtgtatttggtggtgatgattcattgatattgtttgatcagggatacataatttccgacccatgccggcaacttgccggtacttggaatcttgaatgtaaagtgttcgacttcaagtaccccgcattttgcggtaaatttctgctgtgcatagatggaaaatatcagtttgttccagatgcggcaaaatttatcacaaaattaggtagaactgatgtgagagatgtggaagttttgagtgagatttacatctctatcaatgacaattacaaatcttacaaagactttaaggtgcttgatgctttggataaggctttagtggacagatatcgatccccttatagtgctatttctgctttggtttctttatgttatcatatctttgactttaataagtttaagttgctgtttaattgtgaagggaaatttgtggataagaagctgagaaaggacttcgagtggtgaactctaggtcctgatgtttaaatctactgtatttaccttcgcatgatggctactttctcttgtgtgtgttgtggtacctcaactacaagtacttactgtggtaagagatgtgagcgaaagcatgtatattctgaaacaagaaataagagattggaactttacaagaagtatctattggaaccgcaaaaatgcgccctgaatgaaatcgttggtcatagttgtggaatgctatgctccattgcggaagaggcttgtgatcaactgccaatcgtgagtaggttctgtggccaaaagcatgcggatctacatgattcacttctgaaacgttctgaacaggagttacttcttgaatttctccagaagaagatgcaggagctgaaactttctcatatcgtaaaaatggctaagcttgaaagtgaggttaacgcgatacgtaagtccgtagcttcttcttttgaagattctgttggatgtgatgattcttcttccgtttctaagttgtaactAGGTGAAGCAGAAGCTTAAGCGTTTTAGAGCTAGAAATAGCAAGTTAAAATAAGGCTAGTCCGTTATCAACTTGAAAAAGTGGCACCGAGTCGGTGC**TTTTTTT**aaaaaaaaaaaaatgtttgatcagatcattcaaatctgatggtgcccatcaaccatatgatgggagtgtttgcaagtccactataatcgaacttgaaaacgatgcctgaattggaaaccatgaatcttaacggattctggagagaaaatttaggaattggtatgtaagctacaacttccggtagctgcgtcacactttaagagtgtgcatactgagccgaagctcagcttcggtcccccaagggaagacca

**Sequence of RNAγ-gNbPDS-mGFP5.** Sequence in red indicates the sgRNA spacer, sequence in blue indicates the scaffold.

GTATAGCTTGAGCATTACCGTCGTGTAATTGCAACACTTGGCTTGCCAAATAACGCTAAAGCGTTCACGAAACAAACAACACTTCGGCATGGATGTTGTGAAGAAATTCGCCGTCATGTCAGTGACTGTAGTAGCAGGTCCCGTCCTTACGCTTTCATCACCTGTGGTGGTGACGTTTGGAACAGGCTTAATTGCCGTATCTTTGGTGAAACGGTTGCTACAGGAACAACCCCGTGTAATTGCTCACGATCACGAACATTACCCAGGTGGTTCTGAGAGCAGTTCTAGCTCTTGTGCTACCGCGCCTATTTTACGTAATCTTTCGCGAGATCAGTGCGATTCAGAGAATATTGGATGCAGTTCTAGCGCCTGTTCTCCGTCTGAAATTGTGAAAGTTACAAGGCAGGTAGTGGGAGTTGAACGTGGTCTTTACCGGGACATTTTTCAGGACAACGAAATCCCATCAGTCATGGAAGAGAAACTGCAGAAACTCCTTTACTCTGAGGGTGAGAAGATTCGAAGACGTTGCCAATTTGAAGCATCAACGATGCACTCACGCAAAGTAAAGGTTCCGGAGGTAGGTACTATCCCAGATATCCAAACTTGGTTCGATGCTACGTTTCCTGGTAACTCCGTTAGGTTTTCTGATTTCGACGGTTATACTGTTGCTACGGAGGACATTAACATGGATGTTCAGGATTGTAGACTTAAGTTCGGGAAGACTTTTCGACCTTATGAATTTAAGGAATCACTGAAACCAGTACTGAGGACAGCAATGCCAGAAAAACGACAGGGTAGTTTGATTGAAAGTGTGCTGGCCTTTCGTAAAAGAAATTTGGCTGCGCCCAGATTACAAGGAGCTTTGAATGAATGGCACACAATTGAGAATGTGCTAACGAAGGCGTTAAAGGTATTCTTCTTTGAAGATTTAATTGATCGAACGGATCACTGCACTTACGAGTCAGCGCTCAGATGGTGGGATAAACAATCAGTGACAGCTCGAGCGCAGCTCGTGGCGGATCAGCGGAGGTTATGTGATGTTGACTTCACGACTTATAACTTCATGATAAAAAATGATGTAAAGCCGAAGTTAGATCTAACACCTCAAGTTGAATATGCAGCTTTGCAGACTGTTGTATATCCTGATAAGATAGTCAATGCTTTCTTTGGTCCGATCATAAAGGAGATTAATGAACGGATCATCAGAGCGCTTAGACCTCATGTGGTCTTTAATTCTCGTATGACTGCTGATGAACTGAATGAAACAGCTGCCTTTTTGACACCTCATAAGTACAGAGCCTTAGAGATTGATTTTTCAAAATTTGATAAATCAAAGACTGGGCTTCATATCAAAGCTGTCATTGGACTCTATAAGCTCTTTGGCCTAGATGGCCTGTTAAAAGTGCTCTGGGAAAAATCGCAATATCAGACTTACGTGAAAGATAGAAACTTCGGTCTCGAGGCATATCTATTGTATCAGCAAAAGTCAGGAAATTGTGACACTTACGGTTCGAACACCTGGTCTGCCGCCTTGGCGTTGTTAGATTGTCTTCCTTTGGAAGATGCACATTTCTGTGTATTTGGTGGTGATGATTCATTGATATTGTTTGATCAGGGATACATAATTTCCGACCCATGCCGGCAACTTGCCGGTACTTGGAATCTTGAATGTAAAGTGTTCGACTTCAAGTACCCCGCATTTTGTGGTAAATTTCTGCTGTGCATAGATGGAAAATATCAATTTGTTCCAGATGCGGCAAAATTTATCACAAAATTAGGTAGAACTGATGTGAGAGATGTAGAAGTTTTGAGTGAGATTTATATCTCTATCAATGACAATTACAAATCTTACAAAGACTTTAAGGTGCTTGATGCTTTGGATAAGGCTTTAGTGGATAGATATCGATCCCCTTATAGTGCTATTTCTGCTTTGGTTTCTTTATGTTATCATATCTTTGACTTTAATAAGTTTAAGTTGCTGTTTAATTGTGAAGGGAAATTTGTGGATAAGAAGCTGAGAAAAGACTTCGAGTGGTGAACTCTAGGTCCTGATGTTTAAATCTACTGTATTTACCTTCGCATGATGGCTACTTTCTCTTGTGTGTGTTGTGGTACCTTAACTACAAGTACTTACTGTGGTAAGAGATGTGAGCGAAAGCATGTATATTCTGAAACAAGAAATAAGAGATTGGAACTTTACAAGAAGTATCTATTGGAACCGCAAAAATGCGCCCTGAATGGAATCGTTGGACACAGTTGTGGAATGCCATGCTCCATTGCGGAAGAGGCTTGTGATCAACTGCCAATCGTGAGTAGGTTCTGTGGCCAAAAGCATGCGGATCTGTATGATTCACTTCTGAAACGTTCTGAACAGGAGTTACTTCTTGAATTTCTCCAGAAGAAGATGCAGGAGCTGAAACTTTCTCATATCGTAAAAATGGCTAAGCTTGAAAGTGAGGTTAACGCAATACGTAAGTCCGTAGCTTCTTCTTTTGAAGATTCTGTTGGATGTGATGATTCTTCTTCCGTTTCTAAGtaaTTTGGTAGTAGCGACTCCATGTTTTAGAGCTAGAAATAGCAAGttAAAATAAGGCTAGTCCGTTATCAACTTGAAAAAGTggCACCGAGTCGGTGc**tttttTT**GATACCCAGATCATATGAAGgttTTAGAGCTAGAAATAGCAAGTTAAAATAAGGCTAGTCCGTTATCAACTTGAAAAAGTGGCACCGAGTCGGTGC**TTTTTTT**AAAAAAAAAAAAATGTTTGATCAGATCATTCAAATCTGATGGTGCCCATCAACCATATGATGGGAGTGTTTGCAAGTCCACTATAATCGAACTTGAAAACGATGCCTGAATTGGAAACCATGAATCTTAACGGACTCTGGAGAGAAAATTTAGGAATTGGTATGTAAGCTACAACTTCCGGTAGCTGCGTCACACTTTAAGAGTGTGCATACTGAGCCGAAGCTCAGCTTCGGTCCCCCAAGGGAAGACCA

**Diagram and sequence of RNAγ-SmR.** Sequence in purple indicates the *SmR* gene, sequence in blue indicates the scaffold, and the underlined sequences indicate the *Sap*I recognition sites.


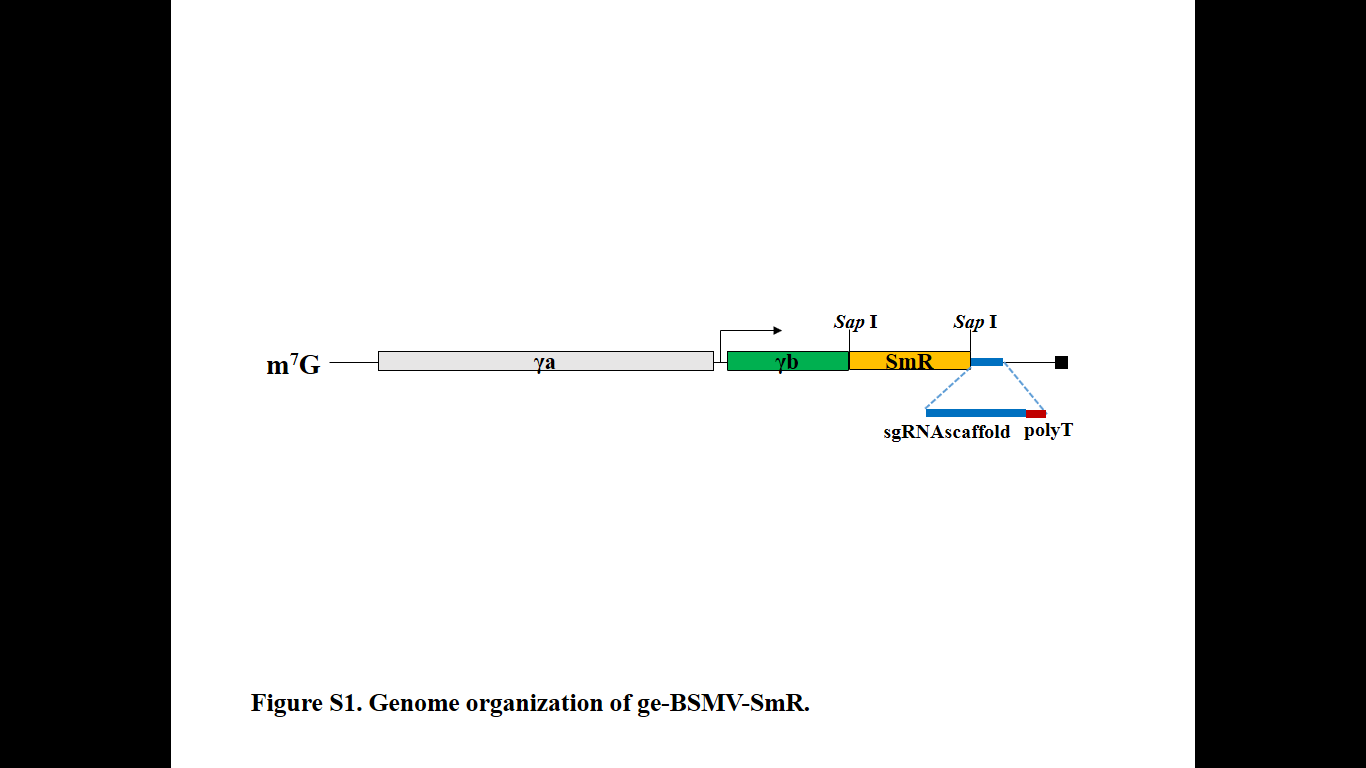


GTATAGCTTGAGCATTACCGTCGTGTAATTGCAACACTTGGCTTGCCAAATAACGCTAAAGCGTTCACGAAACAAACAACACTTCGGCATGGATGTTGTGAAGAAATTCGCCGTCATGTCAGTGACTGTAGTAGCAGGTCCCGTCCTTACGCTTTCATCACCTGTGGTGGTGACGTTTGGAACAGGCTTAATTGCCGTATCTTTGGTGAAACGGTTGCTACAGGAACAACCCCGTGTAATTGCTCACGATCACGAACATTACCCAGGTGGTTCTGAGAGCAGTTCTAGCTCTTGTGCTACCGCGCCTATTTTACGTAATCTTTCGCGAGATCAGTGCGATTCAGAGAATATTGGATGCAGTTCTAGCGCCTGTTCTCCGTCTGAAATTGTGAAAGTTACAAGGCAGGTAGTGGGAGTTGAACGTGGTCTTTACCGGGACATTTTTCAGGACAACGAAATCCCATCAGTCATGGAAGAGAAACTGCAGAAACTCCTTTACTCTGAGGGTGAGAAGATTCGAAGACGTTGCCAATTTGAAGCATCAACGATGCACTCACGCAAAGTAAAGGTTCCGGAGGTAGGTACTATCCCAGATATCCAAACTTGGTTCGATGCTACGTTTCCTGGTAACTCCGTTAGGTTTTCTGATTTCGACGGTTATACTGTTGCTACGGAGGACATTAACATGGATGTTCAGGATTGTAGACTTAAGTTCGGGAAGACTTTTCGACCTTATGAATTTAAGGAATCACTGAAACCAGTACTGAGGACAGCAATGCCAGAAAAACGACAGGGTAGTTTGATTGAAAGTGTGCTGGCCTTTCGTAAAAGAAATTTGGCTGCGCCCAGATTACAAGGAGCTTTGAATGAATGGCACACAATTGAGAATGTGCTAACGAAGGCGTTAAAGGTATTCTTCTTTGAAGATTTAATTGATCGAACGGATCACTGCACTTACGAGTCAGCGCTCAGATGGTGGGATAAACAATCAGTGACAGCTCGAGCGCAGCTCGTGGCGGATCAGCGGAGGTTATGTGATGTTGACTTCACGACTTATAACTTCATGATAAAAAATGATGTAAAGCCGAAGTTAGATCTAACACCTCAAGTTGAATATGCAGCTTTGCAGACTGTTGTATATCCTGATAAGATAGTCAATGCTTTCTTTGGTCCGATCATAAAGGAGATTAATGAACGGATCATCAGAGCGCTTAGACCTCATGTGGTCTTTAATTCTCGTATGACTGCTGATGAACTGAATGAAACAGCTGCCTTTTTGACACCTCATAAGTACAGAGCCTTAGAGATTGATTTTTCAAAATTTGATAAATCAAAGACTGGGCTTCATATCAAAGCTGTCATTGGACTCTATAAGCTCTTTGGCCTAGATGGCCTGTTAAAAGTGCTCTGGGAAAAATCGCAATATCAGACTTACGTGAAAGATAGAAACTTCGGTCTCGAGGCATATCTATTGTATCAGCAAAAGTCAGGAAATTGTGACACTTACGGTTCGAACACCTGGTCTGCCGCCTTGGCGTTGTTAGATTGTCTTCCTTTGGAAGATGCACATTTCTGTGTATTTGGTGGTGATGATTCATTGATATTGTTTGATCAGGGATACATAATTTCCGACCCATGCCGGCAACTTGCCGGTACTTGGAATCTTGAATGTAAAGTGTTCGACTTCAAGTACCCCGCATTTTGTGGTAAATTTCTGCTGTGCATAGATGGAAAATATCAATTTGTTCCAGATGCGGCAAAATTTATCACAAAATTAGGTAGAACTGATGTGAGAGATGTAGAAGTTTTGAGTGAGATTTATATCTCTATCAATGACAATTACAAATCTTACAAAGACTTTAAGGTGCTTGATGCTTTGGATAAGGCTTTAGTGGATAGATATCGATCCCCTTATAGTGCTATTTCTGCTTTGGTTTCTTTATGTTATCATATCTTTGACTTTAATAAGTTTAAGTTGCTGTTTAATTGTGAAGGGAAATTTGTGGATAAGAAGCTGAGAAAAGACTTCGAGTGGTGAACTCTAGGTCCTGATGTTTAAATCTACTGTATTTACCTTCGCATGATGGCTACTTTCTCTTGTGTGTGTTGTGGTACCTTAACTACAAGTACTTACTGTGGTAAGAGATGTGAGCGAAAGCATGTATATTCTGAAACAAGAAATAAGAGATTGGAACTTTACAAGAAGTATCTATTGGAACCGCAAAAATGCGCCCTGAATGGAATCGTTGGACACAGTTGTGGAATGCCATGCTCCATTGCGGAAGAGGCTTGTGATCAACTGCCAATCGTGAGTAGGTTCTGTGGCCAAAAGCATGCGGATCTGTATGATTCACTTCTGAAACGTTCTGAACAGGAGTTACTTCTTGAATTTCTCCAGAAGAAGATGCAGGAGCTGAAACTTTCTCATATCGTAAAAATGGCTAAGCTTGAAAGTGAGGTTAACGCAATACGTAAGTCCGTAGCTTCTTCTTTTGAAGATTCTGTTGGATGTGATGATTCTTCTTCCGTTTCTAAGTAACTAA**GAAGAGC**atgggggaagcggtgatcgccgaagtatcgactcaactatcagaggtagttggcgtcatcgagcgccatctcgaaccgacgttgctggccgtacatttgtacggctccgcagtggatggcggcctgaagccacacagtgatattgatttgctggttacggtgaccgtaaggcttgatgaaacaacgcggcgagctttgatcaacgaccttttggaaacttcggcttcccctggagagagcgagattctccgcgctgtagaagtcaccattgttgtgcacgacgacatcattccgtggcgttatccagctaagcgcgaactgcaatttggagaatggcagcgcaatgacattcttgcaggtatcttcgagccagccacgatcgacattgatctggctatcttgctgacaaaagcaagagaacatagcgttgccttggtaggtccagcggcggaggaactctttgatccggttcctgaacaggatctatttgaggcgctaaatgaaaccttaacgctatggaactcgccgcccgactgggctggcgatgagcgaaatgtagtgcttacgttgtcccgcatttggtacagcgcagtaaccggcaaaatcgcgccgaaggatgtcgctgccgactgggcaatggagcgcctgccggcccagtatcagcccgtcatacttgaagctagacaggcttatcttggacaagaagaagatcgcttggcctcgcgcgcagatcagttggaagaatttgtccactacgtgaaaggcgagatcaccaaggtagtcggcaaataa**GCTCTTC**GGTTTTAGAGCTAGAAATAGCAAGTTAAAATAAGGCTAGTCCGTTATCAACTTGAAAAAGTGGCACCGAGTCGGTGC**TTTTTTT**AAAAAAAAAAAAATGTTTGATCAGATCATTCAAATCTGATGGTGCCCATCAACCATATGATGGGAGTGTTTGCAAGTCCACTATAATCGAACTTGAAAACGATGCCTGAATTGGAAACCATGAATCTTAACGGACTCTGGAGAGAAAATTTAGGAATTGGTATGTAAGCTACAACTTCCGGTAGCTGCGTCACACTTTAAGAGTGTGCATACTGAGCCGAAGCTCAGCTTCGGTCCCCCAAGGGAAGACCA

**Diagram and partial sequence of pCB301-BSMVγ-MCS.** Sequence in black indicates the RNAγ, the underlined sequences indicate the multiple cloning sites.


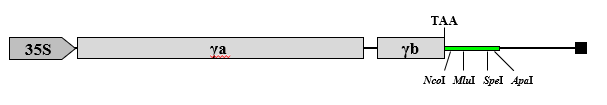


GTATAGCTTGAGCATTACCGTCGTGTAATTGCAACACTTGGCTTGCCAAATAACGCTAAAGCGTTCACGAAACAAACAACACTTCGGCATGGATGTTGTGAAGAAATTCGCCGTCATGTCAGTGACTGTAGTAGCAGGTCCCGTCCTTACGCTTTCATCACCTGTGGTGGTGACGTTTGGAACAGGCTTAATTGCCGTATCTTTGGTGAAACGGTTGCTACAGGAACAACCCCGTGTAATTGCTCACGATCACGAACATTACCCAGGTGGTTCTGAGAGCAGTTCTAGCTCTTGTGCTACCGCGCCTATTTTACGTAATCTTTCGCGAGATCAGTGCGATTCAGAGAATATTGGATGCAGTTCTAGCGCCTGTTCTCCGTCTGAAATTGTGAAAGTTACAAGGCAGGTAGTGGGAGTTGAACGTGGTCTTTACCGGGACATTTTTCAGGACAACGAAATCCCATCAGTCATGGAAGAGAAACTGCAGAAACTCCTTTACTCTGAGGGTGAGAAGATTCGAAGACGTTGCCAATTTGAAGCATCAACGATGCACTCACGCAAAGTAAAGGTTCCGGAGGTAGGTACTATCCCAGATATCCAAACTTGGTTCGATGCTACGTTTCCTGGTAACTCCGTTAGGTTTTCTGATTTCGACGGTTATACTGTTGCTACGGAGGACATTAACATGGATGTTCAGGATTGTAGACTTAAGTTCGGGAAGACTTTTCGACCTTATGAATTTAAGGAATCACTGAAACCAGTACTGAGGACAGCAATGCCAGAAAAACGACAGGGTAGTTTGATTGAAAGTGTGCTGGCCTTTCGTAAAAGAAATTTGGCTGCGCCCAGATTACAAGGAGCTTTGAATGAATGGCACACAATTGAGAATGTGCTAACGAAGGCGTTAAAGGTATTCTTCTTTGAAGATTTAATTGATCGAACGGATCACTGCACTTACGAGTCAGCGCTCAGATGGTGGGATAAACAATCAGTGACAGCTCGAGCGCAGCTCGTGGCGGATCAGCGGAGGTTATGTGATGTTGACTTCACGACTTATAACTTCATGATAAAAAATGATGTAAAGCCGAAGTTAGATCTAACACCTCAAGTTGAATATGCAGCTTTGCAGACTGTTGTATATCCTGATAAGATAGTCAATGCTTTCTTTGGTCCGATCATAAAGGAGATTAATGAACGGATCATCAGAGCGCTTAGACCTCATGTGGTCTTTAATTCTCGTATGACTGCTGATGAACTGAATGAAACAGCTGCCTTTTTGACACCTCATAAGTACAGAGCCTTAGAGATTGATTTTTCAAAATTTGATAAATCAAAGACTGGGCTTCATATCAAAGCTGTCATTGGACTCTATAAGCTCTTTGGCCTAGATGGCCTGTTAAAAGTGCTCTGGGAAAAATCGCAATATCAGACTTACGTGAAAGATAGAAACTTCGGTCTCGAGGCATATCTATTGTATCAGCAAAAGTCAGGAAATTGTGACACTTACGGTTCGAACACCTGGTCTGCCGCCTTGGCGTTGTTAGATTGTCTTCCTTTGGAAGATGCACATTTCTGTGTATTTGGTGGTGATGATTCATTGATATTGTTTGATCAGGGATACATAATTTCCGACCCATGCCGGCAACTTGCCGGTACTTGGAATCTTGAATGTAAAGTGTTCGACTTCAAGTACCCCGCATTTTGTGGTAAATTTCTGCTGTGCATAGATGGAAAATATCAATTTGTTCCAGATGCGGCAAAATTTATCACAAAATTAGGTAGAACTGATGTGAGAGATGTAGAAGTTTTGAGTGAGATTTATATCTCTATCAATGACAATTACAAATCTTACAAAGACTTTAAGGTGCTTGATGCTTTGGATAAGGCTTTAGTGGATAGATATCGATCCCCTTATAGTGCTATTTCTGCTTTGGTTTCTTTATGTTATCATATCTTTGACTTTAATAAGTTTAAGTTGCTGTTTAATTGTGAAGGGAAATTTGTGGATAAGAAGCTGAGAAAAGACTTCGAGTGGTGAACTCTAGGTCCTGATGTTTAAATCTACTGTATTTACCTTCGCATGATGGCTACTTTCTCTTGTGTGTGTTGTGGTACCTTAACTACAAGTACTTACTGTGGTAAGAGATGTGAGCGAAAGCATGTATATTCTGAAACAAGAAATAAGAGATTGGAACTTTACAAGAAGTATCTATTGGAACCGCAAAAATGCGCCCTGAATGGAATCGTTGGACACAGTTGTGGAATGCCATGCTCCATTGCGGAAGAGGCTTGTGATCAACTGCCAATCGTGAGTAGGTTCTGTGGCCAAAAGCATGCGGATCTGTATGATTCACTTCTGAAACGTTCTGAACAGGAGTTACTTCTTGAATTTCTCCAGAAGAAGATGCAGGAGCTGAAACTTTCTCATATCGTAAAAATGGCTAAGCTTGAAAGTGAGGTTAACGCAATACGTAAGTCCGTAGCTTCTTCTTTTGAAGATTCTGTTGGATGTGATGATTCTTCTTCCGTTTCTAAGTAA**CCATGGACGCGTACTAGTGGGCCC**TAAAAAAAAAAAAAAATGTTTGATCAGATCATTCAAATCTGATGGTGCCCATCAACCATATGATGGGAGTGTTTGCAAGTCCACTATAATCGAACTTGAAAACGATGCCTGAATTGGAAACCATGAATCTTAACGGACTCTGGAGAGAAAATTTAGGAATTGGTATGTAAGCTACAACTTCCGGTAGCTGCGTCACACTTTAAGAGTGTGCATACTGAGCCGAAGCTCAGCTTCGGTCCCCCAAGGGAAGACCA
